# Recoverable Detection of Dichloromethane by MEMS Gas Sensor Based on Mo and Ni Co-Doped SnO_2_ Nanostructure

**DOI:** 10.3390/s25092634

**Published:** 2025-04-22

**Authors:** Mengxue Xu, Yihong Zhong, Hongpeng Zhang, Yi Tao, Qingqing Shen, Shumin Zhang, Pingping Zhang, Xiaochun Hu, Xingqi Liu, Xuhui Sun, Zhenxing Cheng

**Affiliations:** 1 Institute of NBC Defence, Beijing 102205, China; xmx_tgcfbwjj@163.com (M.X.); hongpeng_zhang@sina.com (H.Z.); hhuuxxcc@163.com (X.H.); chemlxq@126.com (X.L.); 2 Jiangsu Key Laboratory for Carbon-Based Functional Materials & Devices, Institute of Functional Nano & Soft Materials (FUNSOM), Soochow University, 199 Ren’ai Road, Suzhou 215123, China; 20214014031@stu.suda.edu.cn (Y.Z.); taoyi@suda.edu.cn (Y.T.); qqshen@suda.edu.cn (Q.S.); 3 Suzhou Huiwen Nanotechnology, Co., Ltd., Suzhou 215004, China; smzhang@idmsensor.com (S.Z.); ppzhang@idmsensor.com (P.Z.)

**Keywords:** MEMS gas sensor, dichloromethane, sensing performance, in situ infrared spectroscopy, statistical mechanics, sensing mechanism

## Abstract

**Highlights:**

**What are the main findings?**

- The problem of dichloromethane “poisoned” MOS materials was well solved.
- Gas sensing mechanism of dichloromethane on the MOS material was elucidate.

**What is the implication of the main finding?**

- The best performance of significant response and excellent recoverability by the MEMS sensor to dichloromethane is attributed to a synergetic effect from Mo and Ni co-doping into SnO_2_.
- Kinetically, the transportation of dichloromethane molecules onto the surface determines the response process. Thermodynamically, the output steady electronic signal of the MEMS sensor follows the equations newly derived by statistical mechanics.

**Abstract:**

The challenging problem of chlorine “poisoning” SnO_2_ for poorly recoverable detection of dichloromethane has been solved in this work. The materials synthesized by Ni or/and Mo doping SnO_2_ were spread onto the micro-hotplates (<1 mm^3^) to fabricate the MEMS sensors with a low power consumption (<45 mW). The sensor based on Mo·Ni co-doped SnO_2_ is evidenced to have the best sensing performance of significant response and recoverability to dichloromethane between 0.07 and 100 ppm at the optimized temperature of 310 °C, in comparison with other sensors in this work and the literature. It can be attributed to a synergetic effect of Mo·Ni co-doping into SnO_2_ as being supported by characterization of geometrical and electronic structures. The sensing mechanism of dichloromethane on the material is investigated. In situ infrared spectroscopy (IR) peaks identify that the corresponding adsorbed species are too strong to desorb, although it has demonstrated a good recoverability of the material. A probable reason is the formation rates of the strongly adsorbed species are much slower than those of the weakly adsorbed species, which are difficult to form significant IR peaks but easy to desorb, thus enabling the material to recover. Theoretical analysis suggests that the response process is kinetically determined by molecular transport onto the surface due to the free convection from the concentration gradient during the redox reaction, and the output steady voltage thermodynamically follows the equation only formally identical to the Langmuir–Freundlich equation for physisorption but is newly derived from statistical mechanics.

## 1. Introduction

Metal oxide semiconductor (MOS)-based gas sensors own the advantages of simple device structure, good stability, and excellent gas sensing performances [1,2,3,4,5,6,7] and have been widely applied to detect harmful gases such as CO, NO_2_, H_2_S, CH_4_, HCHO, etc. [8,9,10]. Chemical warfare agents (CWAs) have intrinsically high toxicity. Some of them can be delivered as gases, which cause serious threats to the public [11,12]. Therefore, their detection by gas sensing technology is crucial for maintaining public safety. However, there are some difficulties in carrying out such studies because actual chemical warfare agents are forbidden to be utilized in any routine laboratory where there is a lack of qualifications, protective equipment, and professional operators. In response to this problem, the simulants with much lower toxicity have been widely used in the reported studies due to their similar structures to chemical warfare agents [13,14,15,16,17,18,19], and dichloromethane (DCM) was usually used as a simulant of the phosgene warfare agent that has extremely high lung toxicity [20,21] (Figure S1). In addition, DCM is also widely used in industrial fields, such as industrial solvents, intermediates, and coolants, which was also in the list of Class 2A carcinogens by the International Agency for Research on Cancer of the World Health Organization. The detection of DCM in low concentration is critical in terms of practical applications.

SnO_2_ is a typical n-type MOS material. Although SnO_2_-based sensors have attracted great attention in detecting CWA simulants [13,14,22,23] due to the advantages of high electron mobility, high sensitivity, and good chemical and thermal stability, there are few studies on the detection of DCM. In addition, the limited sensors in the literature also suffer from the disadvantages of poor recovery ability [24,25,26,27], which may be probably due to the poisoning effect on the MOS surface by Cl-containing functional groups in DCM. There has still been a great challenge to develop MOS sensors with excellent sensing performances for DCM. Modification of MOS materials with the investigation of the sensing mechanism could be an important approach to solving the above problem.

The strategy of transition metal doping has been widely applied in the modification of SnO_2_. For example, Lin et al. [28] synthesized a Ni-doped SnO_2_ material by a two-step hydrothermal treatment method whose gas sensitivity to alcohols, aldehydes, and ketones VOCs was improved. In addition, the authors proposed that it is because Ni^2+^ replaces Sn^4+^ in the lattice to generate additional oxygen vacancy, and Ni^2+^ plays the role of a catalytic site to promote the oxidation reaction on the surface. Wang et al. [29] prepared a Mo-doped SnO_2_ nanotubes-based gas sensor that shows increased response, faster response, and recovery rate for ethanol sensing. The authors believed that Mo doping inhibited grain growth so that the nanotubes composed of small nanoparticles (NPs) were thinner and denser, which could provide more adsorption sites. In addition, the network structure formed by the interlaced nanotubes was also more conducive to gas diffusion. In addition to single-element doping, two-element co-doping is also an effective modification technique. For example, Guo et al. [30] improved the electrochemical property of SnO_2_ by co-doping with Ni and sulfur, Ji et al. [31] proposed a co-doping strategy with Sn and Ni to improve the sensing performance of p-type Co_3_O_4_ for acetone, and it was considered to be the result of the abundant asymmetric oxygen vacancy defects induced by co-doping. Most interestingly, an investigation on the SnO_2_-based sensor showed an augmentation of the sensing response from 1.2% up to 50% in detecting DCM of 0.5 ppm at 350 °C once an impregnation of 3 wt.% NiO onto SnO_2_ and a physical mixture with 3 wt.% MoO_3_ have been completed, and it was believed that the NiO promoter enhanced the sensing response level while the MoO_3_ improved the recovery ability [26].

Traditional gas sensors are usually fabricated by depositing MOS sensing materials on a ceramic tube or alumina substrate with a device size of around 10 to 20 mm and a power consumption between 200 and 1000 mW. Recently, the micro-hotplates (MHPs)-based on the Micro-Electro-Mechanical System (MEMS) process have been developed as the heating substrate for gas sensors with a small size (<1 mm) and low power consumption (<100 mW) [32].

This work is devoted to developing the MOS-based MEMS gas sensor for DCM gas with excellent performance by solving the problem of DCM “poisoned” MOS materials with Mo and Ni co-doped SnO_2_ and further elucidating the sensing mechanism as well as the sensing kinetics and thermodynamics. Four kinds of non-doped, Ni-doped, and Mo-doped, as well as Mo·Ni co-doped SnO_2_ materials were synthesized by a simple one-step hydrothermal method and characterized by such different techniques as X-ray diffraction spectroscopy (XRD), transmission electron microscopy (TEM), scanning electron microscopy (SEM), energy-dispersive spectroscopy (EDS), and X-ray photoelectron spectroscopy (XPS). The obtained materials were deposited onto MHPs to fabricate the MEMS sensors and carry out their performance on DCM gas in a wide concentration range under an optimized working condition. An investigation of the surface species from DCM gas adsorbed onto Mo·Ni co-doped SnO_2_ by in situ IR was accomplished at working temperature. The sensing response rate was also discussed with the kinetics of physical adsorption, and the sensing output voltage equivalent to the corresponding electrical current was particularly analyzed by statistical mechanics.

## 2. Materials and Methods

### 2.1. Synthesis and Characterization of SnO_2_-Based Materials

One series of SnO_2_-based materials was prepared from SnCl_2_·2H_2_O (A.R.), (NH_4_)_6_Mo_7_O_24_·4H_2_O (99.9%, Shanghai Macklin Biochemical Technology Co., Ltd., Shanghai, China), and Ni(NO_3_)_2_·6H_2_O (A.R., Shanghai Aladdin Biochemical Technology Co., Shanghai, China) by a simple one-step hydrothermal method (Figure S2). The final products were recorded as pure SnO_2_, Ni-SnO_2_, Mo-SnO_2_ and Mo·Ni-SnO_2_, corresponding to samples No. 1–No. 4 in Table S1, respectively. Each portion of the raw materials used for synthesis contains 40 mL of deionized water and 1.128 g of SnCl_2_·2H_2_O, corresponding to 5 mmol of Sn atoms. When synthesizing Ni-SnO_2_, 161.5 mg of Ni(NO_3_)_2_·6H_2_O was added additionally, which corresponds to approximately 0.55 mmol of Ni atoms. The molar amount of Ni accounts for approximately 10% of the total molar amount of Ni and Sn atoms. As for Mo-SnO_2_, 44.2 mg of (NH_4_)_6_Mo_7_O_24_·4H_2_O was added extra, which corresponds to approximately 0.25 mmol of Mo atoms, with the molar amount of Mo accounting for approximately 5% of the total molar amount of Mo and Sn. When it comes to s Mo·Ni-SnO_2_, 161.5 mg of Ni(NO_3_)_2_·6H_2_O and 44.2 mg of (NH_4_)_6_Mo_7_O_24_·4H_2_O were both added additionally. Firstly, raw materials with the desired amount (Table S1) were added into 40 mL of deionized water and further stirred for 30 min. The obtained precursor solutions were then transferred into a 50 mL Teflon-lined stainless steel autoclave and heated at 180 °C for 12 h; subsequently, the naturally cooled samples were washed with deionized water 3 to 5 times and dried in a vacuum at 60 °C overnight; finally, the resulting solids were thoroughly ground and transferred to a ceramic crucible, heated at 500 °C in a muffle furnace for 4 h at a heating rate of 3 °C/min, and naturally cooled down to room temperature.

The crystal structure and morphology were characterized by XRD (D8 Advance, Bruker, Billerica, MA, USA), SEM (Gemini 300, Zeiss, Jena, Germany), and TEM (Talos F200S, FEI, Lausanne, Switzerland). The element components were analyzed using EDS equipped with SEM and TEM. The composition of the products and electronic state of elements were determined using XPS (EscaLab. 250xi, Thermo Scientific, Waltham, MA, USA), and all XPS spectra were corrected for the C 1s peak at 284.8 eV in advance.

### 2.2. Fabrication and Sensing Measurement of MEMS Sensors to DCM Gas

Fabrication processes of MEMS gas sensors are shown in Figure S3. The Si-based micro-hotplates (MHPs, Suzhou Huiwen Nanotechnology Co., Ltd., Suzhou, China) have a chip size of 0.9 × 0.9 × 0.45 mm in L × W × H. The interdigital electrodes occupy an area in the middle of the MHPs with a size of 140 μm × 140 μm, and the distance between interdigitated electrodes is approximately 7.5 μm. Firstly, the as-prepared SnO_2_ materials were dispersed in ethanol (Polyoxyvinyl ether as a binder, A.R., Shanghai Macklin Biochemical Technology Co., Ltd., Shanghai, China) at a ratio of 100 mg to 100 μL, followed by thoroughly grinding for 10 min to obtain a uniform viscous slurry. The slurry was filled into a capillary tip and then drop-coated onto the interdigitated electrode of MHPs. It is estimated that the amount of material loaded on each MHP would be no more than 0.1 mg. After sintering, wire-bonding, and packaging, MEMS gas sensors were obtained. Figure S3b,c show that a relatively uniform thin film of gas-sensing materials has been formed on MHPs. Figure S3d displays the appearance of the packaged MEMS sensor. It can be seen that the sensors have an extremely small size compared to a coin of 1 RMB, indicating their high potential to be integrated into a portable device.

A static gas distribution system was applied in this work for gas sensing measurements (Figure S4). MEMS sensors connected with an adjustable power supply were placed in a chamber with a volume of about 100 L. Before and after exposure to dichloromethane (DCM, G.R., Beijing Innochem. Technology Co., Ltd., Beijing, China), the chamber was kept open, allowing the sensors to be aged or recovered in ambient air. During any gas sensing measurement, the chamber was closed, and liquid DCM in a certain volume was injected onto the heating plate inside the chamber. Therefore, the injected droplets of DCM could be instantaneously volatilized into gas and diffused to fill in the chamber with a desired concentration [33]. During gas sensing tests, it is found that there is a certain delay between the moment when the DCM liquid droplets were added to the heating plate shown in Figure S4 and the moment when the sensors started to respond. Such a delay is probably due to the vaporization process of the DCM liquid droplets and the diffusion process of the vaporized gas flow from the heating plate to the vicinity of the sensors. Fortunately, the delay time is observed as less than 5 s, which will not cause a significant impact on the implementation of the gas sensing test.

The entire gas sensing measurements were performed in the laboratory, where the room temperature was controlled at 20 ± 5 °C by the air conditioner. While the relative humidity was changed in a wide range of 20–80% RH due to the influence of the climate of the city, Suzhou, where the laboratory is located. The room temperature and relative humidity were measured by the fixed thermo-hygrometer in the laboratory and recorded manually.

### 2.3. Identification of Adsorbed Species from DCM onto Sensing Materials

When it comes to gas sensing mechanisms, some advanced characterization techniques and theoretical approaches have been applied to investigate the interaction of gaseous molecules with the MOS surface, including in situ Raman [34], in situ infrared (IR) spectroscopy [35], in situ transmission electron microscopy [36], theoretical calculations and simulations [37], and gas sensing dynamics studies [38]. Among them, in situ IR has the advantages of a wide spectral range, non-destructivity, and usually presents high sensitivity to chemical composition as well as chemical environment changes in the gas and condensed phase. Therefore, it is widely used in the field of catalysis to monitor the interaction between molecules and catalyst surfaces, which is conducive to learning the reaction mechanism [39].

Herein, the in situ IR spectra of Mo·Ni-SnO_2_ sensing to DCM were measured by in situ IR spectroscopy (Frontier, PerkinElmer, Hong Kong, China) equipped with ZnSe plates in attenuated total reflection (ATR) mode. In ATR mode, the IR light penetrates to a certain depth within the sample and then returns to the surface, during which the incident light is selectively absorbed, causing a change in the intensity of the reflected light. Therefore, the IR absorption information of the sample surface can be obtained, enabling a further analysis of the surface composition and structure of the sample [40].

In a typical test, 80 mg of Mo·Ni-SnO_2_ powder was spread on an aluminum thermostat inside the sample cell of 30 mL. Clean air was introduced firstly at a speed of 50 mL/min; the powder was heated to 310 °C quickly within 1 min as the optimized operating temperature for the Mo·Ni-SnO_2_ sensor, and the background spectrum was collected after 1 h. Subsequently, DCM vapor of 100 ppm was introduced instead of pure air at a speed of 50 mL/min also, and then IR spectra under the DCM atmosphere with the time variation were subtracted from the background spectrum and collected at 2 and 5 min, respectively. Finally, the spectrum was collected every 5 min until it was almost unchanged. During the test, spectra were recorded over the range of 650–4000 cm^−1^ at 1 cm^−1^ resolution.

## 3. Results and Discussion

### 3.1. Geometric and Electronic Structures of SnO_2_-Based Sensing Materials

**Figure 1:**
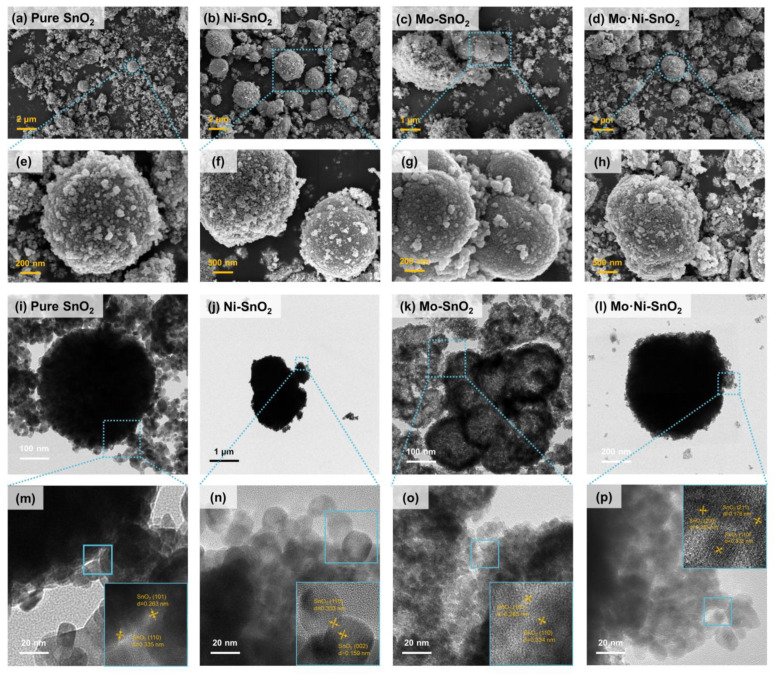
Images of pure SnO_2_, Ni-SnO_2_, Mo-SnO_2_, and Mo·Ni-SnO_2_ (**a**–**d**) SEM, (**e**–**h**) high magnification SEM, (**i**–**l**) TEM, (**m**–**p**) high resolution TEM.

**Figure 2:**
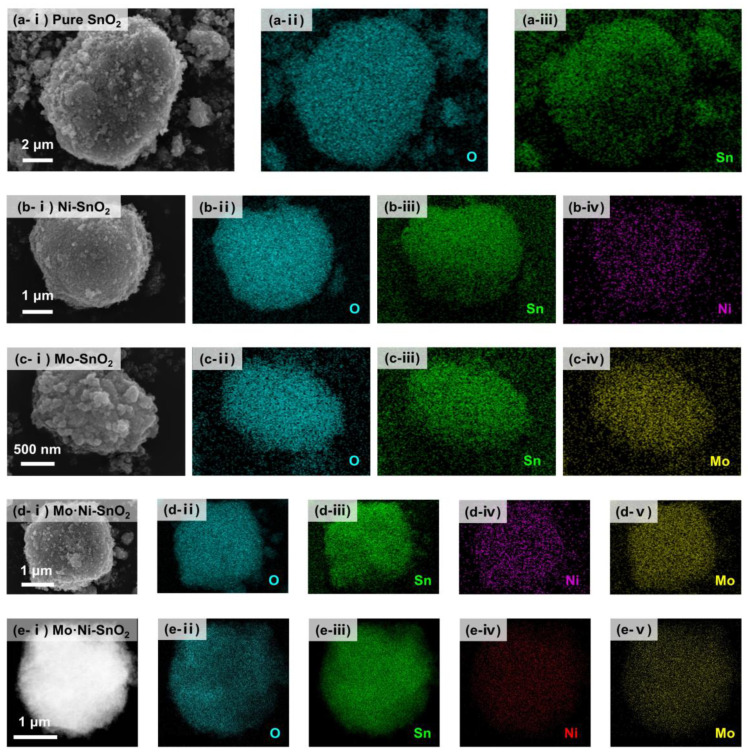
SEM, EDS element mapping images, and EDS spectrum of (**a** (**i**–**iii**)) pure SnO_2_, (**b** (**i**–**iv**)) Ni-SnO_2_, (**c** (**i**–**iv**)) Mo-SnO_2_, and (**d** (**i**–**v**)) Mo·Ni-SnO_2_; (**e** (**i**–**v**)) HRTEM, EDS element mapping images, and EDS spectrum of Mo·Ni-SnO_2_.

For such four sensing materials as pure SnO_2_, Ni-SnO_2_, Mo-SnO_2_ and Mo·Ni-SnO_2_, their morphology and structures were characterized by SEM, TEM (Figure 1), and XRD (Figure S5), and their compositions, distribution, and electronic state were analyzed by EDS (Figure 2 and Figure S6) and XPS (Figure S7).

Pure SnO_2_ is composed of microspheres with a diameter of about 1 μm (Figure 1a) with a coarse surface (Figure 1e) and some dispersed nanoparticles (NPs) with a diameter of about 10–20 nm compactly arranged on the surface of the microspheres (Figure 1i,m). It is possible that the microspheres of pure SnO_2_ are assembled by NPs. Figure 1m–p as insets show the corresponding lattice fringes of four SnO_2_-based materials. The lattice spacings of 0.333 to 0.335 nm, 0.263 to 0.265 nm, 0.235 nm, 0.176 nm, and 0.159 nm can be indexed to the (110), (101), (200), (211), and (002) planes of SnO_2_ with a tetragonal rutile crystal structure, respectively [14,33,41]. This result further suggests that the doped Mo and Ni or their co-doping agent do not significantly affect the crystal structure of SnO_2_.

All four SnO_2_-based materials show similar XRD diffraction peaks (Figure S5) in accordance with the SnO_2_ tetragonal rutile crystal (JPCDS No. 41-1445). The strong peaks at 26.6°, 33.8°, 37.9°, and 51.8° correspond to the crystal planes of (110), (101), (200), and (211), respectively. No diffraction peaks corresponding to other impurities are observed in the XRD pattern of pure SnO_2_, and there are also no diffraction peaks attributed to Ni, Mo, or other compounds containing Ni and Mo. It may be due to a low content of Ni and Mo. In addition, the lattice structure of SnO_2_ would be retained when Ni and Mo are doped into its crystal lattice and substitute Sn [28,29,42,43,44] since the radius of Ni^2+^ (69 pm) is similar to Sn^4+^ (71 pm) and Mo^6+^ (59 pm) slightly smaller than Sn^4+^. The results of XRD patterns (Figure S5) are consistent with the above results from SEM and TEM. The intensity of the correlation diffraction peaks in XRD patterns of Mo-SnO_2_ and Mo·Ni-SnO_2_ are observed to be weaker. Their full widths at half maximum (FWHM) are broader in comparison to those of pure SnO_2_ and Ni-SnO_2_. Moreover, the above differences in the XRD pattern of Mo-SnO_2_ are more significant than that of Mo·Ni-SnO_2_. On one hand, it may be because the ion radii of Mo and Sn are different, and the orderly arrangement of atoms in the original SnO_2_ crystal lattice will be destroyed to cause a certain degree of lattice distortion when the doped Mo partly replaces Sn. As a result, the grain growth is inhibited to broaden the FWHM in their XRD pattern. This is consistent with the inverse relationship between the grain size and the FWHM as Scherer’s formula [45,46,47]. On the other hand, an increase in the FWHM means a decrease in the crystallinity size, which may indicate the formation of defects [29,30,48].

One can learn from Figure 2 and Figure S6 that the signals of Sn and O are very obvious in all EDS mapping images and EDS spectra. Besides, Ni or/and Mo elements are evenly distributed in the SnO_2_-based materials (Figure 2b–e), although the signals of Ni and Mo are relatively weak, possibly because of a low content of doped Mo or/and Ni. The full XPS survey spectra (Figure S7a) show the characteristic peaks corresponding to Sn, O, Mo, and Ni elements. The Sn 3d_5/2_ (487.0 eV) and 3d3/2 (495.4 eV) peaks observed in the XPS spectrum of pure SnO_2_ could be indexed to the Sn^4+^ state (Figure S7b,c). The O 1s peak (530.8 eV) in the XPS spectrum of pure SnO_2_ could be well corresponded to the lattice oxygen in SnO_2_ crystals [49]. According to the Mo 3d spectrum of Mo-SnO_2_ (Figure S7d), the peaks at 233.2 eV and 236.3 eV are assigned to Mo 3d_5/2_ and Mo 3d_3/2_ of Mo^6+^, respectively [50]. Peaks at 232.1 eV and 235.1 eV may be indexed to the Mo^5+^ state [51,52]. Similar results can be obtained from the Mo 3d spectrum of Mo·Ni-SnO_2_ (Figure S7d). In the Ni 2p spectrum of Ni-SnO_2_ (Figure S7e), the peaks located at 854.1 eV and 856.1 eV corresponding to Ni 2p_3/2_ reveal the existence of Ni^2+^, while other peaks are assigned to satellites (860.1 eV, 864.4 eV) [53,54,55]. However, it must be pointed out that the signals of Ni 2p (Figure S7e) are very weak, indicating a low content of doped Ni, being consistent with the EDS result.

Compared with pure SnO_2_, the binding energies of Sn 3d_5/2_ and Sn 3d_3/2_ for Ni-SnO_2_ (Figure S7b) are negatively shifted by about 0.2 eV, indicating that Sn sites trap electrons so that both the oxidation state and coordination number of Sn decrease. The binding energy of O 1s for Ni-SnO_2_ (Figure S7c) is also negatively shifted about 0.2 eV compared to that of pure SnO_2_, which may be due to the slightly smaller electronegativity of Ni (1.91) than that of Sn (1.96). Therefore, the doped Ni leads to a decrease of the sum of the electronegativity of atoms attached to O and an increase of the electron density in the outer shell of O so that the shielding effect becomes stronger, resulting in a decrease of electron binding energy in the inner shell. Moreover, the shift of the binding energy of Sn 3d and O 1s further indicates the doping of Ni in SnO_2_-based material, rather than the formation of NiO/SnO_2_ complex [42,53].

On the contrary, the binding energy of Sn 3d_5/2_ and Sn 3d_3/2_ for Mo-SnO_2_ (Figure S7b) positively shifts by about 0.10 eV compared with pure SnO_2_, indicating that Sn loses electrons, and its oxidation state increases. The binding energy of O 1s for Mo-SnO_2_ (Figure S7c) is also positively shifted by about 0.10 eV, which may be because the larger electronegativity of Mo (2.16) leads to a weaker shielding effect of electrons in the outer shell of O, so that the electron binding energy in the inner shell increases. The above results support a successful doping of Mo in SnO_2_-based material [29,56,57]. Mo·Ni-SnO_2_ (Figure S7b,c) shows the same shift tendency of binding energy as Ni-SnO_2_. These results would indicate an effect of the doped Ni more significant than that of the Mo and Ni co-doping into SnO_2_.

In addition, the O 1s peaks for the four samples (Figure S7c) can be divided into three parts belonging to the lattice oxygen (>70%, O_L_), oxygen vacancy (<20%, O_V_), and surface-chemisorbed oxygen (<10%, O_C_), respectively [48,58], and their binding energies and proportions are listed in Table S2. It can be seen that the proportion of OV increases in the order of Ni-SnO_2_ > Mo·Ni-SnO_2_ > Mo-SnO_2_ ≈ pure SnO_2_, which may be conducive to an improvement of gas sensitivity of MOS materials because there would be more oxygen adsorption sites on the surface [48].

In a word, the above results can support that there could be a synergetic effect of Ni and Mo co-doping into SnO_2_ on the geometrical and electronic structures of SnO_2_ particles, which is in accordance with the concept of “asymmetric oxygen vacancy site” recently proposed in the catalysis field [59].

### 3.2. Performances of MEMS Sensors to DCM Gas

In order to investigate the application feasibility of the as-fabricated MEMS sensors to DCM, the response (R), sensitivity (S), steady-state response (SR), and recovery capability (RC) are defined as Equations (S1)–(S4). Where V_a_ and V_a_’ are the stable output voltage (V_OUT_) of a loading resistance (10 kΩ) in the testing circuit (Figure S8) when sensors are exposed to the ambient air before and after sensing to DCM, respectively, and V_g_ is the output voltage when the sensors are exposed to DCM, V_s_ is the stable V_g_ that can be reached nearly by the end of the response stage. In addition, the response time (t_res_) and recovery time (t_rec_) are determined as the time required to achieve 90% of the total change of output voltage.

The performances of MOS gas sensors have a strong temperature dependence. In addition to the properties of MOS materials, the adsorption thermodynamics and reaction kinetics of the target gas on the MOS surface are also strongly affected by the operating temperature. Besides, the power consumption of the sensor is also related to the operating temperature, which is generated by the heating voltage (V_H_). Therefore, different heating voltages were supplied to the MEMS sensors to study the corresponding operation temperatures and power consumptions first. The temperatures on the sensing area were monitored by a thermal imager (FOTRIC 360 with a measurement range of 40 °C to 2000 °C, Shanghai Fotric Nanotechnology Co., Ltd., Shanghai, China). The current (I) passing through the MEMS sensors was recorded to calculate the power consumption (${P=V}_{H}\times I$). The operation temperatures under a certain V_H_ for the MEMS sensors increase stepwise from 200 °C to 300 °C as V_H_ increases from 1.3 to 2.1 V (Figure S9). Accordingly, the dominant adsorbed oxygen species on the sensing material surface are $O_{\left(\mathrm{ads}\right)}^{-}$ (100 °C < T < 300 °C) and $O_{\left(\mathrm{ads}\right)}^{2-}$ (T > 300 °C) [60]. The power consumption of four MEMS sensors basically ranges from 10 mW to 50 mW, with the operation temperatures ranging from 180 °C to 400 °C (Table S3 and Figure S9), reflecting a low power advantage of MEMS sensors.

To identify the optimal heating voltage for the four different MEMS sensors, they were exposed to 2 ppm DCM at different V_H_. Apparently, their alteration trend of responses is all volcano-like (Figure S9a–d), which is commonly considered to be related to the diffusion, adsorption, and reaction of DCM molecules and desorption of reaction products on the MOS surface, as well as the inherent properties of sensing materials such as electron concentration and carrier migration rate [48,61,62,63,64]. As a result, the optimal heating voltages (or operation temperatures) for the four different sensing materials, such as pure SnO_2_, Ni-SnO_2_, Mo-SnO_2_ and Mo·Ni-SnO_2_ are determined as 1.55 V (275 °C), 1.4 V (210 °C), 2.0 V (330 °C), and 1.8 V (310 °C), respectively (Table 1).

Subsequently, the performances of the four different MEMS sensors for 2 ppm DCM were investigated at their optimal heating voltages (or temperatures) (Figure S10e–h and Table 1).

It can be seen that pure SnO_2_ shows a low sensitivity of 5.4%. Mo-SnO_2_ just presents a sensitivity of 12.5%, being approximately two times as high as pure SnO_2_. It means that Mo doping may have a slight effect of improving the sensitivity of SnO_2_. However, Ni-SnO_2_ and Mo·Ni-SnO_2_ exhibit sensitivities of 26.6% and 37.9%, respectively, being 4.9 and 7.0 times as high as pure SnO_2_, indicating a more significant improvement in sensitivity, which is possibly because of an effect of doped Ni, which brings an increase in the number of active surface sites [48]. Perhaps due to the doping of Ni with a smaller electronegativity (1.91), Sn with a larger electronegativity (1.96) tends to gain electrons, resulting in a decrease in its oxidation state and thereby a decrease in coordination number. In order to achieve the coordination stability and electrical neutrality, the processes of O_2_ dissociation or participation in surface reactions are promoted to regenerate a large number of new oxygen vacancy sites, providing more active sites for gas sensing and resulting in a higher sensitivity [55,59].

In addition, the t_res_. and t_rec_. for Ni-SnO_2_ (Table 1) are 60 s and 130 s, respectively, both being longer than that of pure SnO_2_ (t_res_ = 28 s, t_rec_ = 16 s). The dramatically worsened recovery performance of Ni-SnO_2_ may also be attributed to a decreased coordination number of Sn caused by doped Ni [59], which leads to a preference of the O_2_ adsorbed on the Ni-SnO_2_ surface to dissociate or participate in redox reactions. It means that the competitive adsorption ability of O_2_ decreases, resulting in the fact that during the recovery stage, the surface active sites occupied by DCM and its related functional groups cannot be rapidly and fully released. The above results would indicate that the doped Ni positively enhances the sensitivity but negatively hinders the recovery of SnO_2_ materials. Therefore, it is necessary to solve the problem of poor recovery ability.

**Figure 3:**
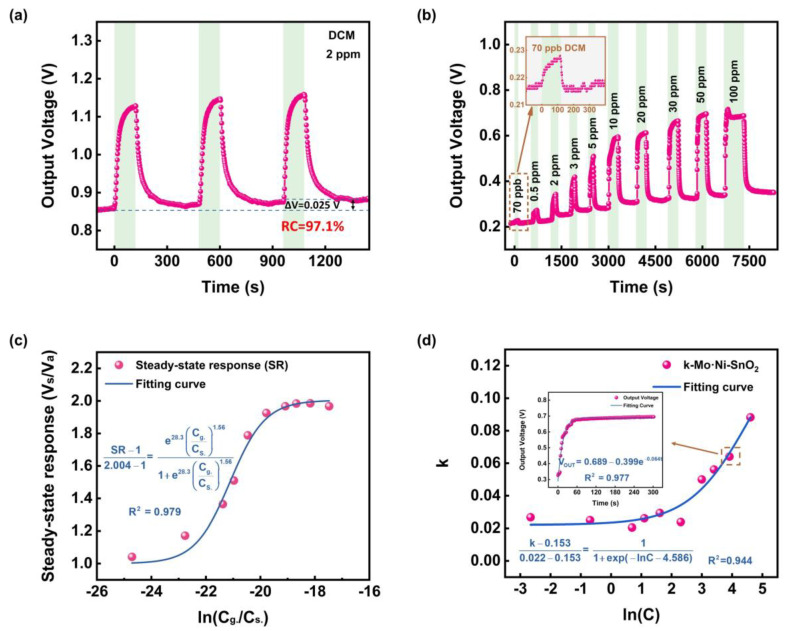
Sensing performances of MEMS sensor Mo·Ni-SnO_2_ to DCM. (**a**) Three cycles of response-recovery curves to 2 ppm DCM. (**b**) Dynamic response curves to DCM under different concentrations. (**c**) Relationship between steady-state response (SR) and ln(C_g_./C_s_.), where C_g_. is the concentration of DCM close to the MOS surface at 310 °C, and C_s_. is the concentration of the virtually saturated vapor at 310 °C for DCM. (**d**) Relationship between kinetic rate constants (k) and the natural logarithm of DCM concentration at room temperature.

The recovery time (t_rec_ = 42 s) for the Mo·Ni-SnO_2_ MEMS sensor to 2 ppm DCM is obviously shorter than that (t_rec_ = 130 s) for Ni-SnO_2_ (Figure S10h and Table 1), and its corresponding recovery capability is close to 97.1% (Figure 3a) due to the Mo-doped effect. Perhaps the coordination numbers of Mo^6+^ and Mo^5+^ (6–8) are higher than that of Sn^4+^ (6), and the oxidation state as well as the coordination number of Sn would increase. In fact, the adsorption of O_2_ onto the oxygen vacancy sites of Mo-doped SnO_2_ materials could probably be promoted to achieve the coordination stability and electrical neutrality [59]; besides, more metal overhang bonds and metal active sites may be generated on the surface due to the doped Mo, which could provide more active chemisorption sites for O_2_ [29]; moreover, Mo could also catalyze the surface adsorption-reaction of oxygen and thereby improve the recovery rate [29]. Since in the catalysis field, the synergistic effect of co-doped Ni and Mo has been interpreted as a possible mechanism that O_2_ molecules are not only easy to bind on, but also to remove from oxygen vacancies on the Mo·Ni-SnO_2_ surface [59], it is therefore possible that the doped Mo is able to promote the formation of chemically adsorbed oxygen species on the Mo·Ni-SnO_2_ surface, which would largely offset the negative effect of doped Ni on the recovery performance. As a consequence, both the response and recovery performances for the Mo·Ni-SnO_2_ sensor to DCM gas are improved.

The above results would indicate that the Mo·Ni-SnO_2_ MEMS sensor exhibits the best overall sensing performance for DCM. Response-recovery curves of Mo·Ni-SnO_2_ to 2 ppm DCM (Figure 3a) do not fluctuate within three cycles, and the recovery capability reaches nearly 97.1%, indicating a good repeatability and reversibility as well. Subsequently, a study will mainly be focused on the investigation of Mo·Ni-SnO_2_ sensing to DCM in the different concentrations from 70 ppb to 100 ppm at 1.8 V (310 °C). One can find that 70 ppb DCM can still be detected with an SR of 1.04, and such a concentration is much lower than 2 ppm as the IDLH (Immediate Damage to Life and Health) for simulating chemical warfare phosgene, and the SR approximately increases linearly at first for the DCM concentration (C_DCM_) less than 10 ppm and then tends to be saturated. Anyway, the above results suggest that the MEMS sensor of Mo·Ni-SnO_2_ has a wide detection range and low actual detection limit, but its sensing capacity tends to be saturated when C_DCM_ becomes higher than 10 ppm.

It should be noted that the baseline drift of the Mo·Ni-SnO_2_ MEMS sensor can be observed from Figure 3b. The probable reasons are as follows [65]. Firstly, the diffusion of dopants in the MOS material as the usage time increases may lead to the change of the local concentration of dopants and thereby the carriers. Secondly, the humidity of the experimental environment in this work is relatively high, which will lead to changes in the sensor baseline. In addition, the gas-sensing material film of the MEMS sensor in this work is formed by sintering as shown in Figure S3. Therefore, long-term work at a high operating temperature, 310 °C, may cause the particles in the gas-sensing film to undergo further sintering, growth, and agglomeration, resulting in an increase in the contact area between particles and a larger carrier transport flux. Generally, methods such as material optimization [66] and compensation by algorithm [67] are tried to alleviate the problem of baseline drift. However, baseline drift remains one of the key factors hindering the development of MOS sensors, and more in-depth research is needed to fundamentally solve it.

Moreover, the response time mostly fluctuates in the range from 30 s to 90 s, while the recovery time does in the range of 15 s to 100 s in the different DCM concentrations from 70 ppb to 100 ppm and is basically less than 50 s for C_DCM_ lower than 5 ppm (Figure S11a–d). It is worth mentioning that the sensor still has a recovery capability of 97.0% and a recovery time of only about 100 s even for C_DCM_ as high as 100 ppm (Figure S11b). These results demonstrate the excellent response and recovery performances for the Mo·Ni-SnO_2_ MEMS sensor.

However, it should be noted that the Mo·Ni-SnO_2_-based MEMS sensor obtained in this work has the shortcoming of poor stability and selectivity. Firstly, as can be seen from Figure S13a, the sensor has poor moisture resistance. This may be because the H_2_O molecules in a high-humidity environment will bind to the active sites on the MOS surface, releasing electrons to the conduction band and causing a decrease in the base resistance of the material. Moreover, the formed hydroxyl groups will occupy the active sites, ultimately leading to a decrease in the sensitivity of the material to DCM [68,69,70]. In addition to the influence of the high relative humidity, the poor stability of the sensor may also be due to drawbacks of the inherent characteristics of the MOS materials and the sensor manufacturing process [71]. The problem of poor stability can be solved to some extent by optimizing the material, adding a porous protective layer upon the MOS surface to enhance its ability to overcome hydroxyl poisoning, or developing a humidity drift compensation model [37,72,73]. Figure S13b shows that the sensor has no significant selectivity for DCM. This may be because the active sites on the surface of the Mo·Ni-SnO_2_ material are not specific to DCM [74]. The selectivity of the sensor for DCM can be improved by adjusting the material composition, modifying the morphology and structure, and introducing molecular sieves, etc. [75]. However, the poor selectivity of a single MOS material may be difficult to effectively solve in a short period. Therefore, constructing a sensor array and combining it with a pattern recognition algorithm is also one of the important strategies to achieve specific recognition of different gases [76].

**Table 1:** Comparison of sensing performances of DCM sensors reported in the literature and in this work.

Material	Device	T ^a^ (°C)	P ^b^ (mW)	Detection Range (ppm)	Sensing Performances				
					C_DCM_ ^c^ (ppm)	S (%)	t_res_ ^d^ (s)	t_rec_ ^e^ (s)	RC ^f^ (%)
SnO_2_ [24]	Alumina flat substrate	350	NA ^g^	0.02~0.8	0.5	29	~600 *	~1700 *	<20 *
SnO_2_-ZnO [77]		350	NA	0.5~1.5	1	60	NA **	NA **	NA **
SnO_2_ [78]		300	NA	0.5~3	0.5	20	NA **	NA **	NA **
SnO_2_-Al_2_O_3_ [78]		300	NA	0.5~3	0.5	8	NA **	NA **	NA **
SnO_2_-In_2_O_3_ [78]		300	NA	0.5~3	0.5	12	NA **	NA **	NA **
SnO_2_-ZrO_2_ [78]		300	NA	0.5~3	0.5	60	NA **	NA **	NA **
SnO_2_-ZnO [78]		300	NA	0.5~3	0.5	66	NA **	NA **	NA **
SnO_2_-Pt [78]		300	NA	0.5~3	0.5	25	NA **	NA **	NA **
SnO_2_ [26]		350	NA	0.1~0.8	0.5	30	~400 *	>2500 *	<5 *
SnO_2_-MoO_3_ [26]		350	NA	0.1~0.8	0.5	22	~150 *	~1000 *	~90 *
SnO_2_-NiO-MoO_3_ [26]		350	NA	0.1~0.8	0.5	50	~100 *	~400 *	>95 *
SnO_2_ [27]		350	NA	0.1~0.8	0.5	17	~500 *	~1000 *	~50 *
SnO_2_-Sm_2_O_3_ [25]		250	NA	0.1~10	10	3470	~600 *	~1200 *	NA **
Pure SnO_2_ (This work)	Silicon-based MEMS	~275	~25	2	2	5	28	16	~100
Ni-SnO_2_ (This work)		210	16.8	2	2	27	60	130	~96
Mo-SnO_2_ (This work)		330	38.0	2	2	13	62	54	~98
Mo·Ni-SnO_2_ (This work)		310	34.2	0.07~100	2	38	78	42	~97
					100	100	32	102	~97

^a^ Operating temperature. ^b^ Power consumption. ^c^ Concentration of DCM that sensors were exposed to when measuring the sensing performances. ^d^ Response time. ^e^ Recovery time. ^f^ Recovery capability. ^g^ Not available from the literature. * No definite values are given in the literature, and the data listed in the table are estimated values according to the figures given in the literature. ** No response-recovery curve has been given in the literature. No figure that can be used to estimate the corresponding values is available in the literature.

A comparison with the performances of DCM by different MOS sensors in this work and the relevant literature as much as we can find [24,25,26,27,77,78] is listed in Table 1. Firstly, it shows that the as-fabricated MEMS sensor based on Mo·Ni-SnO_2_ exhibits a relatively significant response, a wide detection range, a low actual detection limit, and a fast response and recovery rate. Especially, its excellent recovery capability indicates that the technical problem of DCM easily “poisoning” MOS materials has been well solved. In addition, the MEMS sensor also exhibits good repeatability. Furthermore, MOS sensors reported in the literature are non-MEMS types; comparatively, MEMS sensors developed in this work have the advantages of smaller size and lower power consumption [32]. The overall aforementioned strengths of the MEMS sensor based on Mo·Ni-SnO_2_ suggests a high potential for practical application.

### 3.3. Recoverable Mechanism of MEMS Sensor to DCM Gas

**Figure 4:**
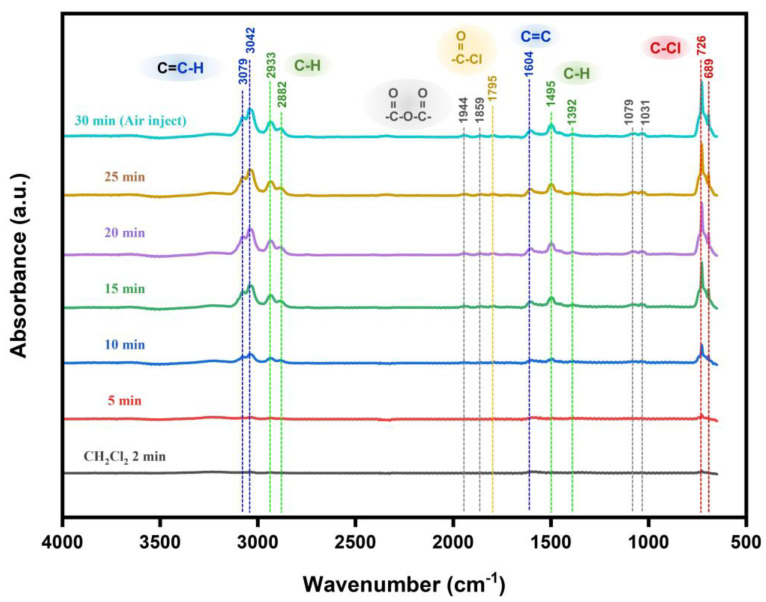
In situ IR spectra of Mo·Ni-SnO_2_ after exposure to 100 ppm DCM with the time variation.

**Figure 5:**
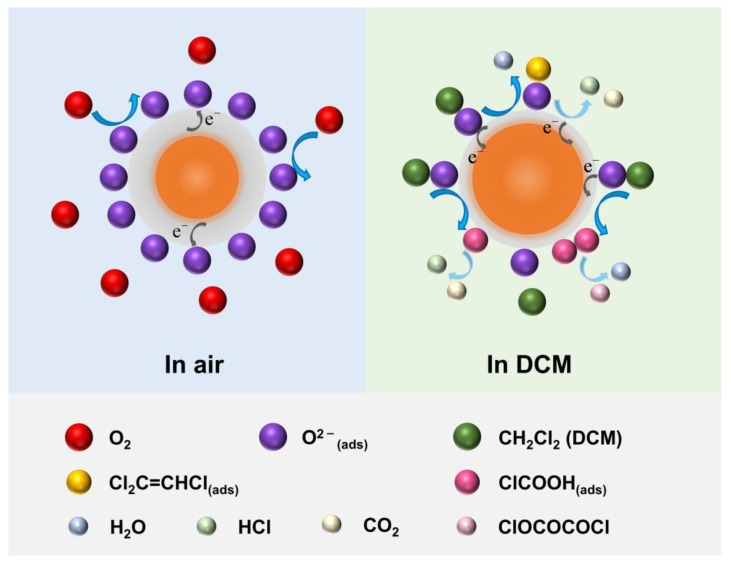
Schematic diagram of the sensing reaction process of DCM onto the Mo·Ni-SnO_2_ surface-based on electron depletion layer (EDL) theory.

The in situ IR spectra of Mo·Ni-SnO_2_ after exposure to 100 ppm DCM at 310 °C with the time variation are presented in Figure 4. The intermediates and products that may generate during the gas sensing reaction at 310 °C are analyzed based on the evolution of the structure and intensity of adsorbed molecular functional groups on the Mo·Ni-SnO_2_ surface. Accordingly, possible mechanisms are proposed for the reactions of DCM with the surface, and the schematic diagram is displayed in Figure 5. To our best knowledge, there is no literature that has reported such a surface reaction.

It can be seen that after an introduction of DCM into the sample cell over 5 min, the strong absorption peaks appear at 3079, 3042, 2933, 2882, 1604, 1495, 1392, 726, and 689 cm^−1^ in the IR spectra. Besides, some weak peaks can be observed at 1944, 1859, 1795, 1079, and 1031 cm^−1^. According to a comparison table of IR absorption characteristic frequencies of various functional groups [79], the absorption peaks at 2933 and 2882 cm^−1^, and 1495 and 1392 cm^−1^ can be attributed to the stretching and bending vibrations of C-H bonds on saturated hydrocarbon groups, respectively. While the peaks at 726 cm^−1^ and 689 cm^−1^ can be attributed to C-Cl stretching vibrations. The above results indicate an adsorption of DCM molecules on the Mo·Ni-SnO_2_ surface, i.e., ${\mathrm{CH}}_{2}{\mathrm{Cl}}_{2\;(\mathrm{ads})}$.

On one hand, the peaks at 3079 cm^−1^ and 3042 cm^−1^ in Figure 4 may be attributed to H atoms connected to an unsaturated C (C-H stretching vibration). The peak at 1604 cm^−1^ can be indexed to the C=C stretching vibration [79]. According to the characteristic peaks and the excellent recovery ability of Mo·Ni-SnO_2_ when sensing DCM, it is reasonable to assume that the products containing C=C bond may be formed during the sensing process. The possible interaction between DCM and adsorbed oxygen on the surface can be derived as follows:

(1) $${2\mathrm{CH}}_{2}{\mathrm{Cl}}_{2\;(\mathrm{ads})}+O_{\left(\mathrm{ads}\right)}^{2-}\to {\mathrm{Cl}}_{2}{C=\mathrm{CHCl}}_{\left(\mathrm{ads}\right)}+\mathrm{HCl}+H_{2}O+2e^{-}$$

(2) $${\mathrm{Cl}}_{2}{C=\mathrm{CHCl}}_{\left(\mathrm{ads}\right)}+H_{2}O+3O_{\left(\mathrm{ads}\right)}^{2-}\to 3\mathrm{HCl}+2{\mathrm{CO}}_{2}+6e^{-}$$

where ${\mathrm{CH}}_{2}{\mathrm{Cl}}_{2\;(\mathrm{ads})}$ on Mo·Ni-SnO_2_ surface is firstly reacted with $O_{\left(\mathrm{ads}\right)}^{2-}$ to form trichloroethylene (${\mathrm{Cl}}_{2}C=\mathrm{CHCl}$), H_2_O, and HCl due to the catalytic effects of transition metal ions at a high temperature. Then, ${\mathrm{Cl}}_{2}C=\mathrm{CHCl}$ further reacts with $O_{\left(\mathrm{ads}\right)}^{2-}$ to form surface HCl and CO_2_. Here, the adsorbed oxygen is taken $O_{\left(\mathrm{ads}\right)}^{2-}$ as an example because the adsorbed oxygen species on the MOS surface are mainly $O_{\left(\mathrm{ads}\right)}^{2-}$ at the optimal heating temperature (310 °C) in this work. The electrons are released back to the conduction band of Mo·Ni-SnO_2_ during the reactions, causing a decrease in the resistance.

On the other hand, the absorption peaks at 1944 and 1859 cm^−1^, 1079 and 1031 cm^−1^ (Figure 4) could be attributed to the asymmetric and symmetric stretching vibration of C=O in anhydride, respectively, and that at 1795 cm^−1^ to the C=O stretching vibration in acid chloride [79]. Accordingly, another possible reaction approach of DCM on the Mo·Ni-SnO_2_ surface could be deduced as follows:

(3) $${\mathrm{CH}}_{2}{\mathrm{Cl}}_{2\;(\mathrm{ads})}+2O_{\left(\mathrm{ads}\right)}^{2-}\to {\mathrm{ClCOOH}}_{\left(\mathrm{ads}\right)}+\mathrm{HCl}+4e^{-}$$

(4) $$2{\mathrm{ClCOOH}}_{\left(\mathrm{ads}\right)}\to \mathrm{ClOC}-O-{\mathrm{COCl}}_{\left(\mathrm{ads}\right)}+H_{2}O$$

(5) $${\mathrm{ClCOOH}}_{\left(\mathrm{ads}\right)}\to \mathrm{HCl}+CO_{2}$$

where ${\mathrm{CH}}_{2}{\mathrm{Cl}}_{2\;(\mathrm{ads})}$ is firstly reacted with $O_{\left(\mathrm{ads}\right)}^{2-}$ to form chloro-formic acid (ClCOOH) and HCl. Subsequently, a reversible opposite reaction may occur between pairwise ClCOOH to remove a molecule of H_2_O and form chloro-formic anhydride (ClOC−O−COCl). Simultaneously, ClCOOH may also further be converted into HCl and CO_2_. The weak intensity of the above four peaks may be because only a small number of DCM molecules have undergone reactions, and the instability of intermediate products may cause difficulty in forming the strong absorption peaks.

However, the IR peak signals attributed to the adsorbed species, such as ${\mathrm{CH}}_{2}{\mathrm{Cl}}_{2\;(\mathrm{ads})}$, ${\mathrm{Cl}}_{2}C=\mathrm{CHCl}$, ClCOOH, and ClOC−O−COCl could not disappear at 310 °C when the Mo·Ni-SnO_2_ material has been exposed again to air even over 30 min. Moreover, the IR peak signals slowly evolve into a steady state with a delayed period higher than 5 min while the sensing output voltage dramatically increases within one minute nearly till to the maximum steady state (Figure 3b) for the DCM concentrations of 100 ppm with a recoverable capacity of the sensor Mo·Ni-SnO_2_ at 310 °C higher than 97%. This would support that thermodynamically there are only very few parts of the species from the surface redox reaction that are too strongly adsorbed to be desorbed from the surface at 310 °C either in DCM gas or pure air, and kinetically their formation is much slower in comparison with the weakly adsorbed and easily desorbed surface species so that there is a recoverable detection of DCM gas by the Mo·Ni-SnO_2_ surface. Therefore, one can propose an overall surface reaction as the recoverable mechanism to detect DCM gas by the Mo·Ni-SnO_2_ surface as follows:

(6) $${\mathrm{CH}}_{2}{\mathrm{Cl}}_{2\;(\mathrm{ads})}+2O_{\left(\mathrm{ads}\right)}^{2-}\to 2\mathrm{HCl}+{\mathrm{CO}}_{2}+4e^{-}$$

where the DCM molecules firstly occupy the Mo·Ni-SnO_2_ surface physically, then interact with adsorbed oxygen chemically, and finally there is a surface redox reaction to generate HCl and CO_2_. Usually, the electrons from the surface redox reaction have been considered to be transferred into the conduction band for decreasing the sensor resistance [80].

It must be pointed out that most of the functional groups corresponding to the IR peaks were attributed to DCM and the intermediate products formed during the reaction after DCM is adsorbed on the MOS surface, rather than to the products of H_2_O, HCl, and CO_2_. This may be because DCM and the intermediate products are more strongly adsorbed, enabling them to form a stable adsorption layer on the MOS surface. However, the adsorption of H_2_O and CO_2_ molecules on the MOS surface at a high temperature of 310 °C may be very weak, leading to a low adsorption amount and a short residence time. It suggests that the molecules probably fail to form a stable adsorption on the surface. Therefore, it is difficult to correspond to significant IR peaks.

When the material was exposed to air again, the occupied active sites on the MOS surface were released mostly, and only the strongly adsorbed intermediate species, including those being identified by IR, were difficult to desorb from the surface. Subsequently, the consumed surface oxygen species are regenerated through grabbing electrons from the conduction band to be transferred towards the oxygen molecules adsorbed from air onto the surface; thus, the sensor resistance increases and gradually returns to the baseline after exposure to air. Particularly, the weakly decaying from the maximum into another steady state for the DCM concentrations of 100 ppm (Figure 3b) seems to be the result of a consumption of surface electrons to transform the physically adsorbed oxygen molecules into surface oxygen species.

However, in order to reveal the gas sensing mechanism of DCM on the surface of the material more systematically and scientifically, further investigations would be carried out in the future by more advanced methods and technologies such as density functional theory calculations [81] and in situ TEM [36].

### 3.4. Kinetics and Thermodynamics of MEMS Sensors to DCM Gas

Recently, the overall kinetic process for a physical adsorption of benzene vapor over active carbon has been evidenced to be a molecular transport non-linearly coupling with a surface-complexing sub-process, and the overall adsorption extent ratio can be expressed by a product of one exponential convex with another logistical growth function [82]. It was the surface-complexing sub-process and the small size of the adsorption system to accelerate the molecular transport and then make the transport extent ratio function degenerate from an error growth function simultaneously due to free convection and diffusion into an exponential convex growth function only due to free convection [82] as being proposed by Levenspiel [83,84].

The kinetic process of the MEMS sensors to DCM gas should theoretically consist of such different sub-processes as molecular transport, surface adsorption, and redox reaction. For the Mo·Ni-SnO_2_-based MEMS sensor to DCM gas in the different concentrations from 70 ppb to 100 ppm at 310 °C under 1.8 V, the response curves shown in Figure 3b could approximately follow an exponential convex growth function as the example being illustrated in the inset of Figure 3d, and the response kinetic rate constants (k) were found to increase with the DCM concentrations, from which one can obtain a Boltzmann growth function as being presented in Figure 3d to probably suggest that the lowest rate constant was limited by DCM self-diffusion coefficient onto surface in air while the highest one was limited by the maximum average internal quantum state number of surface complexes before their redox reaction [85]. Additionally, it has been found in our previous research that the kinetic responses of 24 arrayed MEMS sensors to five different simulants for the CWAs mostly exhibit the exponential convex growth function, whereas rarely does the error growth function with a delayed period not higher than 15 s. These experimental results seem to suggest that it is the molecular transport only by free convection to kinetically determine the rate of physical adsorption with the activation energy (Ea) to be 48 kJ/mol (Figure S12), probably from surface reaction for Mo·Ni-SnO_2_ sensing to DCM and the subsequent sub-processes such as physically complexing, chemically activating, and redox reacting onto the surface very possibly accomplished in an extremely short time.

Thermodynamically, a dependence of the sensing response voltage on C_DCM_ is experimentally observed to follow the equation formally identical to the Langmuir–Freundlich equation for physical adsorption with a power over the C_DCM_ equal to 1.56 (Figure 3c). The total output voltage from the MEMS sensor should consist of two parts: the charge carries from the conduction band in MOS before sensing to DCM and the electrons being released from physical adsorbate molecules back to the conduction band.

When the chemical potential for the electrons in the bulk phase system of MOS materials only in air atmospheres at any temperature is equal to $\mu_{e}$, the distribution of these electrons at any given energy level of $\varepsilon_{i}$ in the system will follow the F-D statistics theory as

(7) $$f_{F.D}=\frac{1}{1+\exp[\beta (\varepsilon_{i}-\mu_{e})]}$$

In the absence of the applied electric field, the number of electrons at all the energy levels in the conduction band must be equal to that of the positive charges to ensure the electric neutrality in the system. Applying a voltage to the sensor system in a steady state, there should be a separation of positive and negative charges in the bulk phase of MOS to increase the average number of electrons by 〈N〉 and then produce the steady-state electrons in an average number of $N_{0}+\langle N\rangle$ and possibly in a saturated number of $2N_{0}$. The non-volume work on each electron is effectively performed by the V applied voltage will be

(8) $$W_{m}^{\prime }=\varepsilon_{i}+\gamma_{eff.}eV$$

where $\gamma_{eff.}$ denotes the effective coefficient for the non-volume work executed by an applied voltage onto each electron in the MOS due to its thermal motion. Thus, the electron distribution at any $\varepsilon_{i}$ energy level becomes

(9a) $$f_{F.D}=\frac{N_{0}+\langle N\rangle }{2N_{0}}=\frac{1}{1+\exp[\beta (\varepsilon_{i}-W_{m}^{\prime }-\mu_{e})]}$$

(9b) $$f_{F.D}=\frac{1}{1+\exp[-\beta (\gamma_{eff.}eV+\mu_{e})]}$$

Equation (9) will give

(10) $$\frac{\langle N\rangle }{N_{0}}=1-\frac{2}{1+\exp[\beta (\gamma_{eff.}eV+\mu_{e})]}$$

Since it is possible that (Supporting Information S3.2)

(11) $$\gamma_{eff.}eV>>\mu_{e}\approx 0$$

one can get

(12) $$\frac{\langle N\rangle }{N_{0}}=\frac{\langle \rho \rangle }{\rho_{0}}=\frac{\langle I\rangle }{I_{0}}=1-\frac{2}{1+\exp(\beta \gamma_{eff.}eV)}$$

Equation (12) is the steady-state conductivity equation for the bulk phase of the MOS material, where ρ and I are the conductive electron number density and steady-state current under an applied voltage of V, respectively, and can be well verified by the electrical current through the semiconductor, such as silicon nano-wires [86]. The electrical resistance for the sensor, when there is an absence of physical adsorption of DCM gas by the Mo·Ni-SnO_2_-based MEMS sensor at 310 °C (Figure 3b), can be calculated from the output voltage to be $R_{S}=190k\Omega$, which can suggest that the $\gamma_{eff.}$ effective coefficient for the non-volume work by an applied voltage onto each electron in the MOS due to its thermal motion should be far from one unit.

For the adsorptive molecule in a system of physical adsorption onto a homogeneous surface by monolayer, the chemical potentials, respectively, for the adsorptive and adsorbate molecules can be expressed as Equation (13a) and (13b) according to the definition of chemical potentials.

(13a) $$\mu_{v./g.}=\mu_{0}(T,p^{\Theta })+k_{B}T\ln(\varphi_{v./g.}\frac{p_{v./g.}}{p^{\Theta }})$$

(13b) $$\mu_{ads.}=\mu_{0}(T,p^{\Theta })+k_{B}T\ln(\varphi_{ads.}\frac{p_{ads.}}{p^{\Theta }})$$

and the non-volume work ($W_{m}^{\prime }$) onto each electron of this adsorbate molecule, at any energy level, executed by the interfacial tension between the adsorptive and adsorbate phase as well as the adsorption force totally resulted from both the attractive and repulsive interaction between the adsorbate and surface can be formulated by

(14a) $$\gamma_{exch.}W_{m}^{\prime }=\gamma_{exch.}\varepsilon_{i}+A_{m}\sigma_{ads.-v./g.}-\mu_{ads.}$$

where $\gamma_{exch.}$ denotes the average number of electrons exchanged with the surface per each adsorbate molecule, and one can consider the interfacial energy between the adsorptive and adsorbate phase, according to the literature [87], as

(14b) $$A_{m}\sigma_{ads.-v./g.}=k_{B}T\ln\frac{\varphi_{ads.}}{\varphi_{v./g.}}$$

The intramolecular electrons at any energy level could follow the Fermi–Dirac distribution and equal the degree of the electrons exchanged between the adsorbate and surface, as

(15a) $$f_{F.D}=\frac{1}{1+\exp[\beta (\varepsilon_{i}-W_{m}^{\prime }-\mu_{e})]}=\frac{\gamma_{exch.}a}{\gamma_{exch.}a_{m}}$$

(15b) $$\mu_{e}=\gamma_{exch.}^{-1}\mu_{v./g.}$$

where a denotes the adsorption amount at the subcritical vapor or supercritical gas pressure of $p_{v./g.}$ and $a_{m}$ is the monolayer adsorption amount. One can get

(15c) $$\varepsilon_{i}-W_{m}^{\prime }-\mu_{e}=-\gamma_{exch.}^{-1}k_{B}T\ln\frac{p_{v./g.}}{p_{ads.}}$$

when

(15d) $$k_{B}T\ln\frac{p_{v./g.}}{p_{ads.}}=k_{B}T\ln\frac{p_{s.}}{p_{ads.}}-k_{B}T\ln\frac{p_{s.}}{p_{v./g.}},p_{ads.}<<p_{v./g.}\leq p_{s.}$$

One can write the first term in the above right-hand side as

(15e) $$k_{B}T\ln\frac{p_{s.}}{p_{ads.}}=\varepsilon >>0$$

where ε is the absolute net value, totally from both the attractive and repulsive interaction between adsorbate-surface, while the second term in the right hand $-k_{B}T\ln(p_{s.}/p_{v./g.})$, is the physical adsorption potential early defined by Polanyi [88], with the pressures of adsorbed vapor or gas ($p_{v./g.}$)and truly or virtually saturated vapor ($p_{s.}$). Finally, one find

(15f) $$\frac{a}{a_{m}}=\frac{1}{1+\exp\{\beta \gamma_{exch.}^{-1}[-\varepsilon -k_{B}T\ln(p_{v./g.}/p_{s.})]\}}$$

The above statistical interpretation of the Langmuir–Freundlich physisorption equation points out the fundamental nature of the surface coverage degree for a physical adsorbate onto a homogeneous surface by a monolayer as the Fermi–Dirac distribution of the electrons in the adsorbate molecules at any given energy level to exchange with the surface.

When there is an application of a voltage such as 1.8 V to the Mo·Ni-SnO_2_-based MEMS sensor onto which there may be some physical adsorbate molecules of DCM possibly by monolayer, the subsequent redox reaction of DCM adsorbate molecules upon surface activation to open the relevant closed electronic shells at high temperatures such as 310 °C will release some of the electrons back to the conduction band and produce the increment in steady-state current to be expressed as

(16a) $$\frac{\langle \Delta I\rangle }{\Delta I_{0}}=\frac{\gamma_{eff.}\gamma_{rel.}a}{\gamma_{eff.}\gamma_{rel.}a_{m}}=\frac{1}{1+\exp\{\beta \gamma_{rel.}^{-1}[-\varepsilon -\gamma_{eff.}\gamma_{rel.}eV-k_{B}T\ln(p_{g.}/p_{s.})]\}}$$

where 〈ΔI〉 denotes the increment of steady-state current, $\gamma_{eff.}$ is the effective coefficient for the non-volume work executed by an applied voltage onto each electron released from the adsorbate upon surface redox reaction, ε will represent the net energy totally resulted, from chemisorption and surface redox reaction while $\gamma_{rel.}$ will do the effective number of electrons released from surface redox reaction per each adsorbate molecule to the electron conduction band. The above Equation (16a) can be reformulated into the equation formally identical to the Langmuir–Freundlich equation for physical adsorption by the steady gas concentration as

(16b) $$\frac{\langle \Delta I\rangle }{\Delta I_{0}}=\frac{a}{a_{m}}=\frac{K{\left(C_{g.}/C_{s.}\right)}^{\gamma_{rel.}^{-1}}}{K{\left(C_{g.}/C_{s.}\right)}^{\gamma_{rel.}^{-1}}+1}$$

(16c) $$K=\exp(\beta \gamma_{rel.}^{-1}\varepsilon +\beta \gamma_{eff.}eV)$$

Equation (16c) can give

(16d) $$\gamma_{rel.}^{-1}\varepsilon +\gamma_{eff.}eV=\frac{\ln K}{\beta }=k_{B}T\ln K$$

One can experimentally obtain the $\gamma_{rel.}^{-1}=1.56$, and K=exp(28.3) values in Figure 3c when the virtually saturated vapor at 310 °C for DCM was calculated from its vapor equation to be $C_{s.}=4.11\;\times \;10^{8}\;\mathrm{ppm}$, one will find

(16e) $$\gamma_{eff.}eV+\gamma_{rel.}^{-1}\varepsilon =1.37eV$$

It has been found in our previous work that amounts of physically adsorbed CH_4_ onto the microporous carbon surface were lower down with increasing the subcritical up to supercritical temperatures; and an application of Equation (16b) to the isotherms could prove the van der Waals interaction energy of ε between the adsorptive and physisorption sites to be a constant valued at 0.083 eV. The sensor response to a given concentration of 2 ppm DCM was experimentally found to firstly increase and then slightly decrease with increasing the sensing temperature from 200 °C to 350 °C (Figure S9) and have the sensing recoverability close to 100%, clearly indicating that the surface physisorption of DCM onto MOS was quite quickly evolved into another chemisorption followed by a reversible surface redox reaction with the $\gamma_{rel.}^{-1}\varepsilon$ values in Equation (16e) remarkably dependent on sensing temperature. At the high concentration of 100 ppm DCM, the sensing recoverability could still be larger than 95%. The reversible surface redox reaction being proposed from the IR study indicates 4 electrons released to the electronic conduction band per each DCM molecule is experimentally found to be 0.64. Hence, this would indicate that the $\gamma_{eff.}$ is equal to 0.64/4 = 16% as the effective coefficient due to thermal motion defined by Equation (9a) for the non-volume work executed by an applied voltage onto each electron released from the chemisorbed molecules onto the metal oxide surface of the MEMS sensor. As a result, the ε value as the net energy totally resulted from chemisorption and surface redox reaction can be calculated to be 1.21 eV.

## 4. Conclusions

The MOS MEMS gas sensors were fabricated with pure, Ni-doped, Mo-doped, and Mo·Ni co-doped SnO_2_ materials simply synthesized via a one-step hydrothermal method on MEMS-based micro-hotplates, respectively. Different characterization techniques such as XRD, TEM, EDS, and XPS could evidence that these four materials are constructed by microspheres assembled from nanoparticles (NPs) with such surface oxygen species as lattice oxygen (>70%), oxygen vacancy (<20%), and chemisorbed oxygen (<10%). The MEMS sensor based on Mo·Ni-SnO_2_ to DCM gas, with the small size (<1 mm^3^) and low power consumption (<45 mW), shows better performance than other DCM sensors reported in the literature, and the technical problem of DCM easily “poisoning” the MOS materials can be well solved because of the synergistic effect of Ni·Mo co-doping into SnO_2_. Under the experimentally optimized operation condition of 310 °C under 1.8 V, the Mo·Ni-SnO_2_ sensor exhibits not only the high voltage response (38%) nearly within 78 s but also the excellent recovery capability (>97%) closely within 42 s, with a wide concentration range from 70 ppb to 100 ppm, whose low detection limit is much lower than 2 ppm as the IDLH (immediate damage to life and health) concentration for the phosgene warfare agent. The synergistic effect of Ni and Mo co-doping into SnO_2_ probably promotes the formation of surface oxygen species and largely offsets the negative effect of Ni doping on the recovery performance. However, it should be noted that the optimization of the amount of doping additives is necessary in future work to improve the sensing performance of the sensors. Moreover, more in-depth work is needed to improve the moisture resistance, stability, and selectivity for DCM of the sensor. Furthermore, the sensing response process can be suggested to be kinetically determined by the molecular transport onto surface in an exponential convex growth function only due to free convection; thermodynamically, the output voltage for sensing response of Mo·Ni-SnO_2_ to DCM at steady-state is experimentally found to follow the equation being newly derived out from statistical mechanics for the steady-state electrical currents and formally identical to the Langmuir–Freundlich for physical adsorption, whose adsorption constant and power over concentration can suggest that the effective coefficient of the electrical work onto each electron released from chemisorption and surface redox reaction is 16% while that from semiconductor is far from unity.

## Data Availability

The original contributions presented in this study are included in the article/Supplementary Material. Further inquiries can be directed to the corresponding author(s).

## Supplementary Materials

The following supporting information can be downloaded at: https://www.mdpi.com/article/10.3390/s25092634/s1, Figure S1: The molecular structures of phosgene (left) and DCM (right); Figure S2: Schematic diagram of synthesizing the micro-hot plate structure in MEMS sensors; Figure S3: Fabricating processes of MEMS sensors (a) Drop-coated the slurry of SnO_2_ materials on MHPs followed by sintering to form a thin film, (b) SEM image and photograph of the MEMS sensor after wire-bonding, (c) Enlarged SEM image of the sensing layer on a MEMS sensor, and (d) The appearance of MEMS sensors after packaging; Figure S4: Schematic diagram of the static gas distribution system; Figure S5: XRD patterns of Pure SnO_2_, Ni-SnO_2_, Mo-SnO_2_, and Mo·Ni-SnO_2_; Figure S6: EDS spectrum with SEM of (a) Pure SnO_2_, (b) Ni-SnO_2_, (c) Mo-SnO_2_, and (d) Mo·Ni-SnO_2_ ; (e) EDS spectrum with HRTEM of Mo·Ni-SnO_2_; Figure S7: XPS spectra (a) Full scan of four materials (Pure SnO_2_, Ni-SnO_2_, Mo-SnO_2_, and Mo·Ni-SnO_2_), (b) Sn 3d of four materials, (c) O 1s of four materials (d) Mo 3d of Mo-SnO_2_, Mo·Ni-SnO_2_, (e) Ni 2p_3/2_ of Ni-SnO_2_, Mo·Ni-SnO_2_; Figure S8: Simplified circuit diagram of the MEMS gas sensor test element; Figure S9: Relationship between the operating temperature and heating voltage of different MEMS sensors (inset is the thermal imaging on a micro-hotplate at a certain heating voltage); Figure S10: (a–d) Relationship between response and heating voltage (e–h). Dynamic response curves of 4 MEMS sensors to 2 ppm DCM at the optimal heating voltage; Figure S11: (a) Response and recovery time of MEMS sensor Mo·Ni-SnO_2_ to 70 ppb DCM, (b) Response time, recovery time, and recovery capability of MEMS sensor Mo·Ni-SnO_2_ to 100 ppm DCM, (c) Relationship between response time and concentration of DCM (70 ppb to100 ppm), (d) Relationship between recovery time and concentration of DCM (70 ppm to 100 ppm); Figure S12: Temperature dependence of linear fitted plots of lnk in the presence of 2 ppm DCM using Mo·Ni-SnO_2_ as sensing materials; Figure S13: Stability and selectivity of MEMS sensor Mo·Ni-SnO_2_ to DCM (a) The stability test results of response of the sensor during 9 days at 310 °C in an environment where the relative humidity fluctuates greatly and (b)Selectivity investigation of the sensor towards 2 ppm DCM, CH_4_, H_2_S, EtOH, dimethyl methylphosphonate (DMMP), 2-chloroethyl ethyl sulfide (CEES), and acetonitrile (ACN) at 310 °C; here DMMP, CEES, and ACN are the simulant agents of chemical warfare agents sarin, mustard gas, and hydrogen cyanide, respectively; Table S1: Raw materials required to synthesize SnO_2_-based materials; Table S2: O 1s peaks in XPS spectra of Pure SnO_2_, Ni-SnO_2_, Mo-SnO_2_, and Mo·Ni-SnO_2_; Table S3: Power consumption of MEMS sensors (Pure SnO_2_, Ni-SnO_2_, Mo-SnO_2_, and Mo·Ni-SnO_2_) under different heating voltages. Reference [89] is cited in the Supplementary Materials.

## Abbreviations

The following abbreviations are used in this manuscript:

MOS	Metal oxide semiconductor
CWAs	Chemical warfare agents
DCM	Dichloromethane
MHPs	Micro-hotplates
MEMS	Micro-Electro-Mechanical System
XRD	X-ray diffraction spectroscopy
TEM	Transmission electron microscopy
SEM	Scanning electron microscopy
EDS	Energy-dispersive spectroscopy
XPS	X-ray photoelectron spectroscopy
IR	Infrared spectroscopy
FWHM	Full widths at half maximum
R	Response
S	Sensitivity
SR	Steady-state response
RC	Recovery capability

## References

[1] Kumar S., Pavelyev V., Mishra P., Tripathi N., Sharma P., Calle F. (2020). A review on 2D transition metal di-chalcogenides and metal oxide nanostructures based NO_2_ gas sensors. Mater. Sci. Semicond. Process..

[2] Kong Y., Li Y., Cui X., Su L., Ma D., Lai T., Yao L., Xiao X., Wang Y. (2022). SnO_2_ nanostructured materials used as gas sensors for the detection of hazardous and flammable gases: A review. Nano Mater. Sci..

[3] Modak M., Rane S., Jagtap S. (2023). WO_3_: A review of synthesis techniques, nanocomposite materials and their morphological effects for gas sensing application. Bull. Mater. Sci..

[4] Bigiani L., Zappa D., Maccato C., Gasparotto A., Sada C., Comini E., Barreca D. (2020). Hydrogen gas sensing performances of p-type Mn_3_O_4_ nanosystems: The role of built-in Mn_3_O_4_/Ag and Mn_3_O_4_/SnO_2_ junctions. Nanomaterials.

[5] Kang Y., Yu F., Zhang L., Wang W., Chen L., Li Y. (2021). Review of ZnO-based nanomaterials in gas sensors. Solid State Ionics.

[6] Masuda Y. (2022). Recent advances in SnO_2_ nanostructure based gas sensors. Sens. Actuators B Chem..

[7] Neri G., Donato N. (2016). Resistive gas sensors. Wiley Encyclopedia of Electrical and Electronics Engineering.

[8] Wicker S., Guiltat M., Weimar U., Hémeryck A., Barsan N. (2017). Ambient humidity influence on CO detection with SnO_2_ gas sensing materials. a combined drifts/DFT investigation. J. Phys. Chem. C.

[9] Li Q., Zeng W., Li Y. (2022). Metal oxide gas sensors for detecting NO_2_ in industrial exhaust gas: Recent developments. Sens. Actuators B Chem..

[10] Zhou S., Wang H., Hu J., Lv T., Rong Q., Zhang Y., Zi B., Chen M., Zhang D., Wei J. (2022). Formaldehyde gas sensor with extremely high response employing cobalt-doped SnO_2_ ultrafine nanoparticles. Nanoscale Adv..

[11] Sezigen S., Kenar L. (2020). Recent sulfur mustard attacks in Middle East and experience of health professionals. Toxicol. Lett..

[12] Vucinic S., Antonijevic B., Tsatsakis A.M., Vassilopoulou L., Docea A.O., Nosyrev A.E., Izotov B.N., Thiermann H., Drakoulis N., Brkic D. (2017). Environmental exposure to organophosphorus nerve agents. Environ. Toxicol. Pharmacol..

[13] Lee M.J., Cheong H.W., Son L.D.N., Yoon Y.S. (2008). Surface reaction mechanism of acetonitrile on doped SnO_2_ sensor element and its response behavior. Jpn. J. Appl. Phys..

[14] Yang Z., Zhang Y., Zhao L., Fei T., Liu S., Zhang T. (2022). The synergistic effects of oxygen vacancy engineering and surface gold decoration on commercial SnO_2_ for ppb-level DMMP sensing. J. Colloid Interface Sci..

[15] Patil L.A., Bari A.R., Shinde M.D., Deo V., Kaushik M.P. (2012). Detection of dimethyl methyl phosphonate—A simulant of sarin: The highly toxic chemical warfare—Using platinum activated nanocrystalline ZnO thick films. Sens. Actuators B Chem..

[16] Yoo R., Cho S., Song M.J., Lee W. (2015). Highly sensitive gas sensor based on Al-doped ZnO nanoparticles for detection of dimethyl methylphosphonate as a chemical warfare agent simulant. Sens. Actuators B Chem..

[17] Yoo R., Yoo S., Lee D., Kim J., Cho S., Lee W. (2017). Highly selective detection of dimethyl methylphosphonate (DMMP) using CuO nanoparticles /ZnO flowers heterojunction. Sens. Actuators B Chem..

[18] Alali K.T., Liu J., Moharram D., Liu Q., Yu J., Chen R., Li R., Wang J. (2020). Fabrication of electrospun Co_3_O_4_/CuO p-p heterojunctions nanotubes functionalized with HFIP for detecting chemical nerve agent under visible light irradiation. Sens. Actuators B Chem..

[19] Bigiani L., Zappa D., Barreca D., Gasparotto A., Sada C., Tabacchi G., Fois E., Comini E., Maccato C. (2019). Sensing nitrogen mustard gas simulant at the ppb scale via selective dual-site activation at Au/Mn_3_O_4_ interfaces. ACS Appl. Mater. Interfaces.

[20] Schwenk M. (2018). Chemical warfare agents. Classes and targets. Toxicol. Lett..

[21] Barreca D., Maccato C., Gasparotto A. (2022). Metal oxide nanosystems as chemoresistive gas sensors for chemical warfare agents: A focused review. Adv. Mater. Interfaces.

[22] Sohn J.R., Park H.D., Lee D.D. (2000). Acetonitrile sensing characteristics and infrared study of SnO_2_-based gas sensors. Appl. Surf. Sci..

[23] Sberveglieri G., Baratto C., Comini E., Faglia G., Ferroni M., Pardo M., Ponzoni A., Vomiero A. (2009). Semiconducting tin oxide nanowires and thin films for chemical warfare agents detection. Thin Solid Films.

[24] Lee W.S., Lee S.C., Lee S.J., Lee D.D., Huh J.S., Jun H.K., Kim J.C. (2005). The sensing behavior of SnO_2_ -based thick-film gas sensors at a low concentration of chemical agent simulants. Sens. Actuators B Chem..

[25] Aliha H.M., Khodadadi A.A., Mortazavi Y. (2013). The sensing behaviour of metal oxides (ZnO, CuO and Sm_2_O_3_) doped-SnO_2_ for detection of low concentrations of chlorinated volatile organic compounds. Sens. Actuators B Chem..

[26] Lee S.C., Choi H.Y., Lee S.J., Lee W.S., Huh J.S., Lee D.D., Kim J.C. (2009). Novel SnO_2_-based gas sensors promoted with metal oxides for the detection of dichloromethane. Sens. Actuators B Chem..

[27] Lee S.C., Kim S.Y., Lee W.S., Jung S.Y., Hwang B.W., Ragupathy D., Lee D.D., Lee S.Y., Kim J.C. (2011). Effects of textural properties on the response of a SnO_2_-based gas sensor for the detection of chemical warfare agents. Sensors.

[28] Lin Z., Li N., Chen Z., Fu P. (2017). The effect of Ni doping concentration on the gas sensing properties of Ni doped SnO_2_. Sens. Actuators B Chem..

[29] Wang L., Ma S., Li J., Wu A., Luo D., Yang T., Cao P., Ma N., Cai Y. (2021). Mo-doped SnO_2_ nanotubes sensor with abundant oxygen vacancies for ethanol detection. Sens. Actuators B Chem..

[30] Guo M., Yang H., Du Y., Fan J. (2021). Influence of Ni and S co-doping of SnO_2_ on its electrochemical performance. Int. J. Electrochem. Sci..

[31] Ji X., Chang J., Deng Z., Shen C., Li M., Wang S., You L., Kumar M., Fang X., Meng G. (2024). Boosting acetone response of p-type Co_3_O_4_ sensor via Sn and Ni co-doping for diabetes diagnosis. Sens. Actuators B Chem..

[32] Guo M., Brewster Ii J.T., Zhang H., Zhao Y., Zhao Y. (2022). Challenges and opportunities of chemiresistors based on microelectromechanical systems for chemical olfaction. ACS Nano.

[33] Meng L., Bu W., Li Y., Qin Q., Zhou Z., Hu C., Chuai X., Wang C., Sun P., Lu G. (2021). Highly selective triethylamine sensing based on SnO/SnO_2_ nanocomposite synthesized by one-step solvothermal process and sintering. Sens. Actuators B Chem..

[34] Akande A.A., Machatine A.G.J., Masina B., Chimowa G., Matsoso B., Roro K., Duvenhage M.-M., Swart H., Bandyopadhyay J., Ray S.S. (2018). Blue- and red-shifts of V_2_O_5_ phonons in NH_3_ environment by *in situ* Raman spectroscopy. J. Phys. D Appl. Phys..

[35] Chu J., Wang Q., Yang A., Pan J., Yuan H., Wang X., Rong M. (2022). MOF-doped WO_3_ nanofibers for the SF_6_ decomposition products: The effects of MOF-modification on the sensitive performance and mechanism. Appl. Surf. Sci..

[36] Wang X., Yao F., Xu P., Li M., Yu H., Li X. (2021). Quantitative structure–activity relationship of nanowire adsorption to SO_2_ revealed by *in situ* TEM technique. Nano Lett..

[37] Chu B., Liu S., Qin Q., Zhao R., Chen K., Hou X., Li R., Li C., Chen J., Dong L. (2023). Surface cleaning and protection engineering via functionalization to awaken the intrinsic catalytic activity of Co_3_O_4_. Adv. Funct. Mater..

[38] Vuong N.M., Kim D., Kim H. (2015). Surface gas sensing kinetics of a WO_3_ nanowire sensor: Part 1—Oxidizing gases. Sens. Actuators B Chem..

[39] Weng X., Yang S., Ding D., Chen M., Wan H. (2022). Applications of in-situ wide spectral range infrared absorption spectroscopy for CO oxidation over Pd/SiO_2_ and Cu/SiO_2_ catalysts. Chin. J. Catal..

[40] Yang S., Zhang Q., Yang H., Shi H., Dong A., Wang L., Yu S. (2022). Progress in infrared spectroscopy as an efficient tool for predicting protein secondary structure. Int. J. Biol. Macromol..

[41] Drzymała E., Gruzeł G., Depciuch J., Budziak A., Kowal A., Parlinska-Wojtan M. (2017). Structural, chemical and optical properties of SnO_2_ NPs obtained by three different synthesis routes. J. Phys. Chem. Solids.

[42] Hu J., Wang T., Wang Y., Huang D., He G., Han Y., Hu N., Su Y., Zhou Z., Zhang Y. (2018). Enhanced formaldehyde detection based on Ni doping of SnO_2_ nanoparticles by one-step synthesis. Sens. Actuators B Chem..

[43] Azam A., Ahmed A.S., Ansari M.S., Shafeeq M M., Naqvi A.H. (2010). Study of electrical properties of nickel doped SnO_2_ ceramic nanoparticles. J. Alloys Compd..

[44] Li W.-T., Zhang X.-D., Guo X. (2017). Electrospun Ni-doped SnO_2_ nanofiber array for selective sensing of NO_2_. Sens. Actuators B Chem..

[45] Scherrer P. (1912). Bestimmung der inneren Struktur und der Größe von Kolloidteilchen mittels Röntgenstrahlen. Kolloidchemie Ein Lehrbuch.

[46] Patterson A.L. (1939). The scherrer formula for X-ray particle size determination. Phys. Rev..

[47] Tirumalareddygari S.R., Guddeti P.R., Ramakrishna Reddy K.T. (2018). A critical study of the optical and electrical properties of transparent and conductive Mo-doped ZnO films by adjustment of Mo concentration. Appl. Surf. Sci..

[48] Huang X., Chen K., Xie W., Li Y., Yang F., Deng Y., Li J., Jiang F., Shu Y., Wu L. (2023). Chemiresistive gas sensors based on highly permeable Sn-doped bismuth subcarbonate microspheres: Facile synthesis, sensing performance, and mechanism study. Adv. Funct. Mater..

[49] Cheng J.P., Wang B.B., Zhao M.G., Liu F., Zhang X.B. (2014). Nickel-doped tin oxide hollow nanofibers prepared by electrospinning for acetone sensing. Sens. Actuators B Chem..

[50] Wang Q., Zhao H., Li F., She W., Wang X., Xu L., Jiao H. (2019). Mo-doped Ni_2_P hollow nanostructures: Highly efficient and durable bifunctional electrocatalysts for alkaline water splitting. J. Mater. Chem. A.

[51] Bica De Moraes M.A., Trasferetti B.C., Rouxinol F.P., Landers R., Durrant S.F., Scarmínio J., Urbano A. (2004). Molybdenum oxide thin films obtained by the hot-filament metal oxide deposition technique. Chem. Mater..

[52] Xie F., Choy W.C.H., Wang C., Li X., Zhang S., Hou J. (2013). Low-temperature solution-processed hydrogen molybdenum and vanadium bronzes for an efficient hole-transport layer in organic electronics. Adv. Mater..

[53] Li Z., Yi J. (2017). Enhanced ethanol sensing of Ni-doped SnO_2_ hollow spheres synthesized by a one-pot hydrothermal method. Sens. Actuators B Chem..

[54] Jian J., Shi Y., Ekeroth S., Keraudy J., Syväjärvi M., Yakimova R., Helmersson U., Sun J. (2019). A nanostructured NiO/cubic SiC p–n heterojunction photoanode for enhanced solar water splitting. J. Mater. Chem. A.

[55] Jin B., Cheng S., Sun Y., Xie P., Li L. (2024). High selectivity toward electrochemical ozone production of Sb-SnO_2_ with Cu and Ni co-doped. Adv. Funct. Mater..

[56] Bozheyev F., Akinoglu E.M., Wu L., Lou S., Giersig M. (2020). Effect of Mo-doping in SnO_2_ thin film photoanodes for water oxidation. Int. J. Hydrogen Energy.

[57] Wang J., Mueller D.N., Crumlin E.J. (2024). Recommended strategies for quantifying oxygen vacancies with X-ray photoelectron spectroscopy. J. Eur. Ceram. Soc..

[58] Xiao W., Jin Z., Yang W., Liu S. (2023). Construction of flower-like hierarchical Ni-doped SnO_2_ nanosheets and their gas sensing properties for ethanol. New J. Chem..

[59] Yu K., Lou L., Liu S., Zhou W. (2020). Asymmetric oxygen vacancies: The intrinsic redox active sites in metal oxide catalysts. Adv. Sci..

[60] Krishna K.G., Umadevi G., Parne S., Pothukanuri N. (2023). Zinc oxide based gas sensors and their derivatives: A critical review. J. Mater. Chem. C.

[61] Korotcenkov G. (2007). Metal oxides for solid-state gas sensors: What determines our choice?. Mater. Sci. Eng. B.

[62] Zhu L.-Y., Yuan K., Yang J.-G., Ma H.-P., Wang T., Ji X.-M., Feng J.-J., Devi A., Lu H.-L. (2019). Fabrication of heterostructured p-CuO/n-SnO_2_ core-shell nanowires for enhanced sensitive and selective formaldehyde detection. Sens. Actuators B Chem..

[63] Zhu L., Miao X., Ou L., Mao L., Yuan K., Sun S., Devi A., Lu H. (2022). Heterostructured α-Fe_2_O_3_ @ZnO@ZIF-8 core–shell nanowires for a highly selective MEMS-based ppb-level H_2_S gas sensor system. Small.

[64] Fu X., Liu J., Wan Y., Zhang X., Meng F., Liu J. (2012). Preparation of a leaf-like CdS micro-/nanostructure and its enhanced gas-sensing properties for detecting volatile organic compounds. J. Mater. Chem..

[65] Holmberg M., Artursson T., Pearce T.C., Schiffman S.S., Nagle H.T., Gardner J.W. (2002). Drift compensation, standards, and calibration methods. Handbook of Machine Olfaction.

[66] Yan W., Xu H., Ling M., Zhou S., Qiu T., Deng Y., Zhao Z., Zhang E. (2021). MOF-derived porous hollow Co_3_ O_4_ @ZnO cages for high-performance MEMS trimethylamine sensors. ACS Sens..

[67] Liang Z., Zhang L., Tian F., Wang C., Yang L., Guo T., Xiong L. (2021). A novel WWH problem-based semi-supervised online method for sensor drift compensation in E-nose. Sens. Actuators B Chem..

[68] Krishna K.G., Parne S., Pothukanuri N., Kathirvelu V., Gandi S., Joshi D. (2022). Nanostructured metal oxide semiconductor-based gas sensors: A comprehensive review. Sens. Actuators A Phys..

[69] Isaac N.A., Pikaar I., Biskos G. (2022). Metal oxide semiconducting nanomaterials for air quality gas sensors: Operating principles, performance, and synthesis techniques. Microchim. Acta.

[70] Chai H., Zheng Z., Liu K., Xu J., Wu K., Luo Y., Liao H., Debliquy M., Zhang C. (2022). Stability of metal oxide semiconductor gas sensors: A review. IEEE Sens. J..

[71] Nadargi D.Y., Umar A., Nadargi J.D., Lokare S.A., Akbar S., Mulla I.S., Suryavanshi S.S., Bhandari N.L., Chaskar M.G. (2023). Gas sensors and factors influencing sensing mechanism with a special focus on MOS sensors. J. Mater. Sci..

[72] Sayegh S., Lee J.H., Yang D.H., Weber M., Iatsunskyi I., Coy E., Razzouk A., Kim S.S., Bechelany M. (2021). Humidity-resistant gas sensors based on SnO_2_ nanowires coated with a porous alumina nanomembrane by molecular layer deposition. Sens. Actuators B Chem..

[73] Yan M., Wu Y., Hua Z., Lu N., Sun W., Zhang J., Fan S. (2021). Humidity compensation based on power-law response for MOS sensors to VOCs. Sens. Actuators B Chem..

[74] Deng Y. (2019). Semiconducting Metal Oxides for Gas Sensing.

[75] Zhang D., Yang Z., Yu S., Mi Q., Pan Q. (2020). Diversiform metal oxide-based hybrid nanostructures for gas sensing with versatile prospects. Coord. Chem. Rev..

[76] Hu W., Wan L., Jian Y., Ren C., Jin K., Su X., Bai X., Haick H., Yao M., Wu W. (2019). Electronic noses: From advanced materials to sensors aided with data processing. Adv. Mater. Technol..

[77] Yun K.-H., Yun K.-Y., Cha G.-Y., Lee B.H., Kim J.C., Lee D.-D., Huh J.S. (2005). Gas sensing characteristics of ZnO-doped SnO_2_ sensors for simulants of the chemical agents. Mater. Sci. Forum.

[78] Choi N.-J., Kwak J.-H., Lim Y.-T., Bahn T.-H., Yun K.-Y., Kim J.-C., Huh J.-S., Lee D.-D. (2005). Classification of chemical warfare agents using thick film gas sensor array. Sens. Actuators B Chem..

[79] Silverstein R.M., Webster F.X., Kiemle D.J., Bryce D.L. (2015). Spectrometric Identification of Organic Compounds.

[80] Ji H., Zeng W., Li Y. (2019). Gas sensing mechanisms of metal oxide semiconductors: A focus review. Nanoscale.

[81] Zhu L.Y., Ou L.X., Mao L.W., Wu X.Y., Liu Y.P., Lu H.L. (2023). Advances in noble metal-decorated metal oxide nanomaterials for chemiresistive gas sensors: Overview. Nano-Micro Lett..

[82] Tang H.M., Zhang H.P., Cheng Z.X. (2024). A new kinetic equation suitable for three different adsorption systems. Sep. Purif. Technol..

[83] Levenspiel O. (1996). The Chemical Reactor Omnibook.

[84] Levenspiel O. (2012). Tracer Technology: Modeling the Flow of Fluids.

[85] Dai X.Z., Zhou H., Zhang H.P., Zhu H.Y., Wang C., Tang H.M., Cheng Z.X. (2024). A new kinetic model on hydrolysis of sulfur mustard non-dissolved and dissolved in aqueous solution. J. Mol. Liq..

[86] Liu X., Zhang H., Huang Z., Cheng Z., Li T. (2023). Highly sensitive detection of sarin simulant by a functional SiNW array. Chem. Pap..

[87] Tian Z.F. (2001). Phase equilibrium theory on the interface layer model of physical interface. J. Colloid Interface Sci..

[88] Polanyi M. (1914). Über die adsorption vom standpunkt des dritten wärmesatzes. Verh. Dtsch. Phys. Ges..

[89] Quinn J.J., Yi K.S. (2018). Solid State Physics: Principles and Modern Applications.

