# Supplementary material for: Recoverable Detection of Dichloromethane by MEMS Gas Sensor Based on Mo and Ni Co-Doped SnO2 Nanostructure
Source: Sensors (Basel). 2025 Apr 22;25(9):2634. doi: 10.3390/s25092634 (PMC12073819; doi:10.3390/s25092634)
Supplement: Supplementary file 1 [file sensors-25-02634-s001.zip › sensors-3544317-supplementary.pdf]

Supporting Information

Recoverable detection of dichloromethane by MEMS gas sensor based on Mo and Ni co-doped SnO_2 nanostructure

Section S1 Tables

Table S1 Raw materials required to synthesize SnO_2 based materials.

Number	Sample name	Amount of raw materials		
		$SnCl_2·2H_2O$	$Ni(NO_3)_2·6H_2O$	$(NH_4)_6Mo_7O_{24}·4H_2O$
No.1	Pure SnO_2	1.128 g (5 mmol Sn)	—	—
No.2	Ni-SnO_2	1.128 g (5 mmol Sn)	161.5 mg (0.55 mmol Ni)	—
No.3	Mo-SnO_2	1.128 g (5 mmol Sn)	—	44.2 mg (0.25 mmol Mo)
No.4	Mo·Ni-SnO_2	1.128 g (5 mmol Sn)	161.5 mg (0.55 mmol Ni)	44.2 mg (0.25 mmol Mo)

*Raw materials with desired amount will be added in 40 mL deionized water.

Table S2 O 1s peaks in XPS spectra of Pure SnO_2, Ni-SnO_2, Mo-SnO_2, and Mo·Ni-SnO_2.

Sample name	Binding energy and proportion		
	O_L	O_V	O_C
Pure SnO_2	530.8 eV, 75.48%	531.8 eV, 13.94%	532.8 eV, 10.57%
Ni-SnO_2	530.6 eV, 71.69%	531.6 eV, 20.09%	532.6 eV, 8.22%
Mo-SnO_2	530.9 eV, 77.19%	531.9 eV, 14.93%	532.9 eV, 7.88%
Mo·Ni-SnO_2	530.7 eV, 73.74%	531.7 eV, 18.43%	532.7 eV, 7.83%

Table S3 Power consumption of MEMS sensors (Pure SnO_2, Ni-SnO_2, Mo-SnO_2, and Mo·Ni-SnO_2) under different heating voltage.

Heating voltage (V_H, V)	Power consumption of MEMS sensors (P, mW)			
	Pure SnO_2	Ni-SnO_2	Mo-SnO_2	Mo·Ni-SnO_2
1.3	20.8	14.3	20.8	20.8
1.4	22.4	16.8	22.4	22.4
1.5	24.0	19.5	24.0	24.0
1.6	25.6	20.8	27.2	25.6
1.7	28.9	23.8	30.6	28.9
1.8	34.2	25.2	34.2	34.2
1.9	36.1	26.6	36.1	36.1
2.0	38.0	30.0	38.0	38.0
2.1	44.1	33.6	42.0	42.0

Section S2 Figures

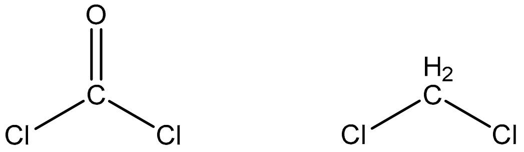

Figure S1 The molecular structures of phosgene (left) and DCM (right).

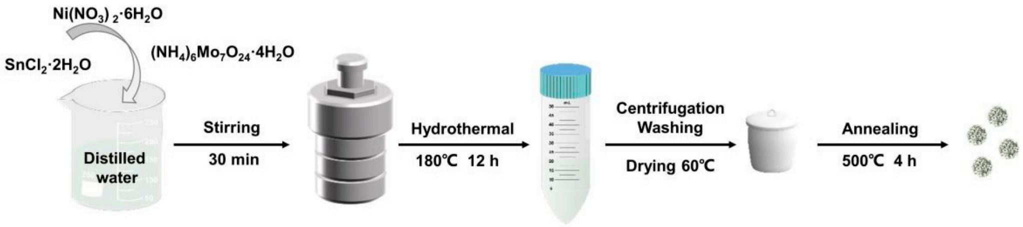

Figure S2 Schematic diagram of synthesizing the micro-hot plate structure in MEMS sensors.

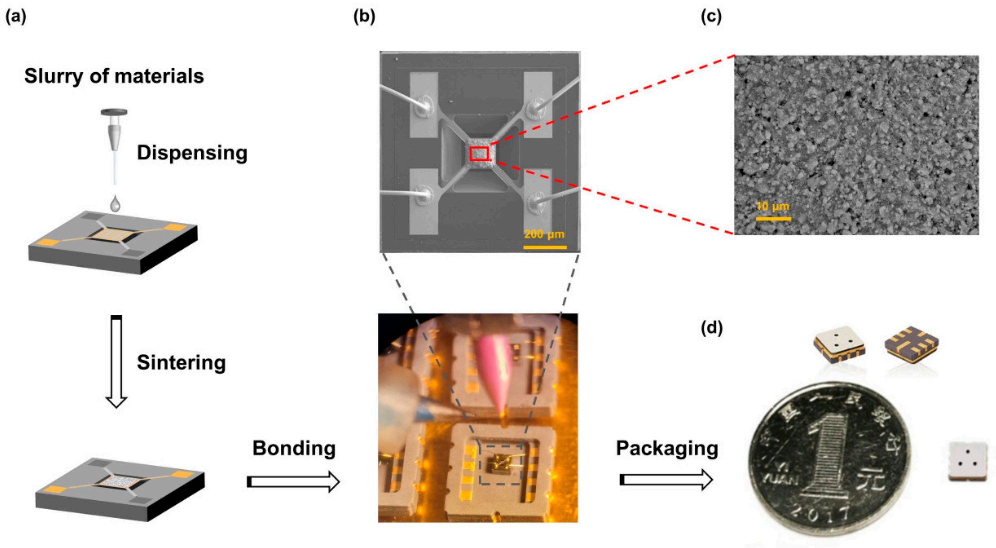

Figure S3 Fabricating processes of MEMS sensors **(a)** Drop-coated the slurry of SnO_2 materials on MHPs followed by sintering to form a thin film, **(b)** SEM image and photograph of the MEMS sensor after wire-bonding, **(c)** Enlarged SEM image of the sensing layer on a MEMS sensor, and **(d)** The appearance of MEMS sensors after packaging.

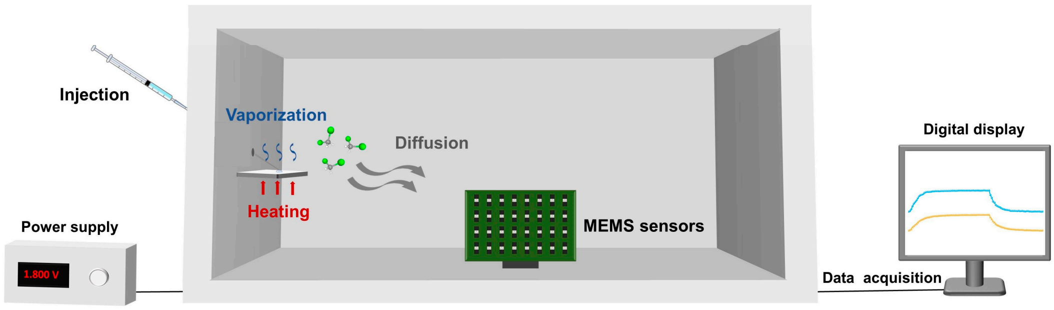

Figure S4 Schematic diagram of the static gas distribution system.

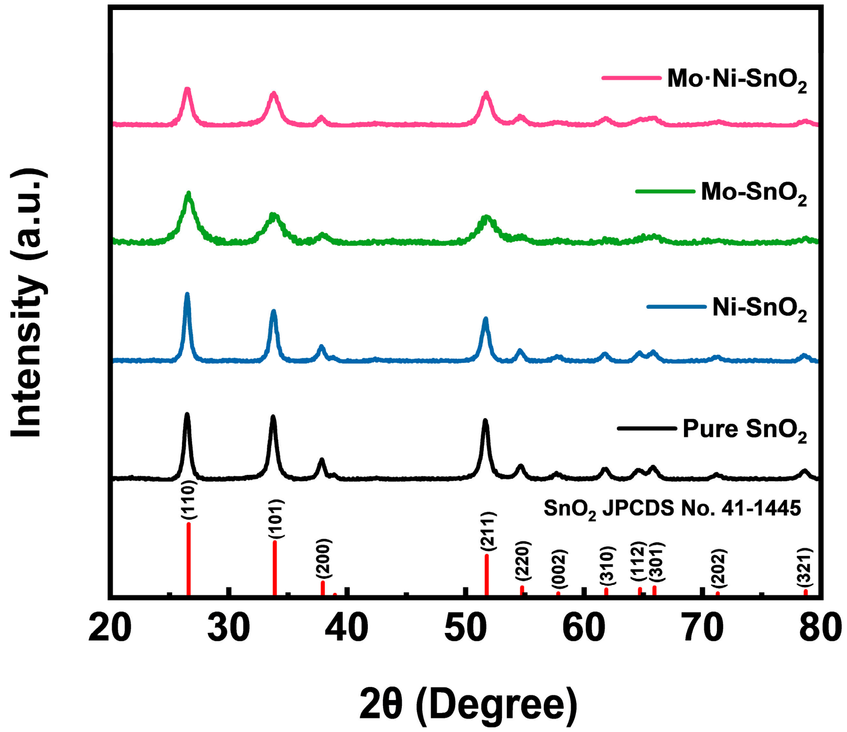

Figure S5 XRD patterns of Pure SnO_2, Ni-SnO_2, Mo-SnO_2, and Mo·Ni-SnO_2.

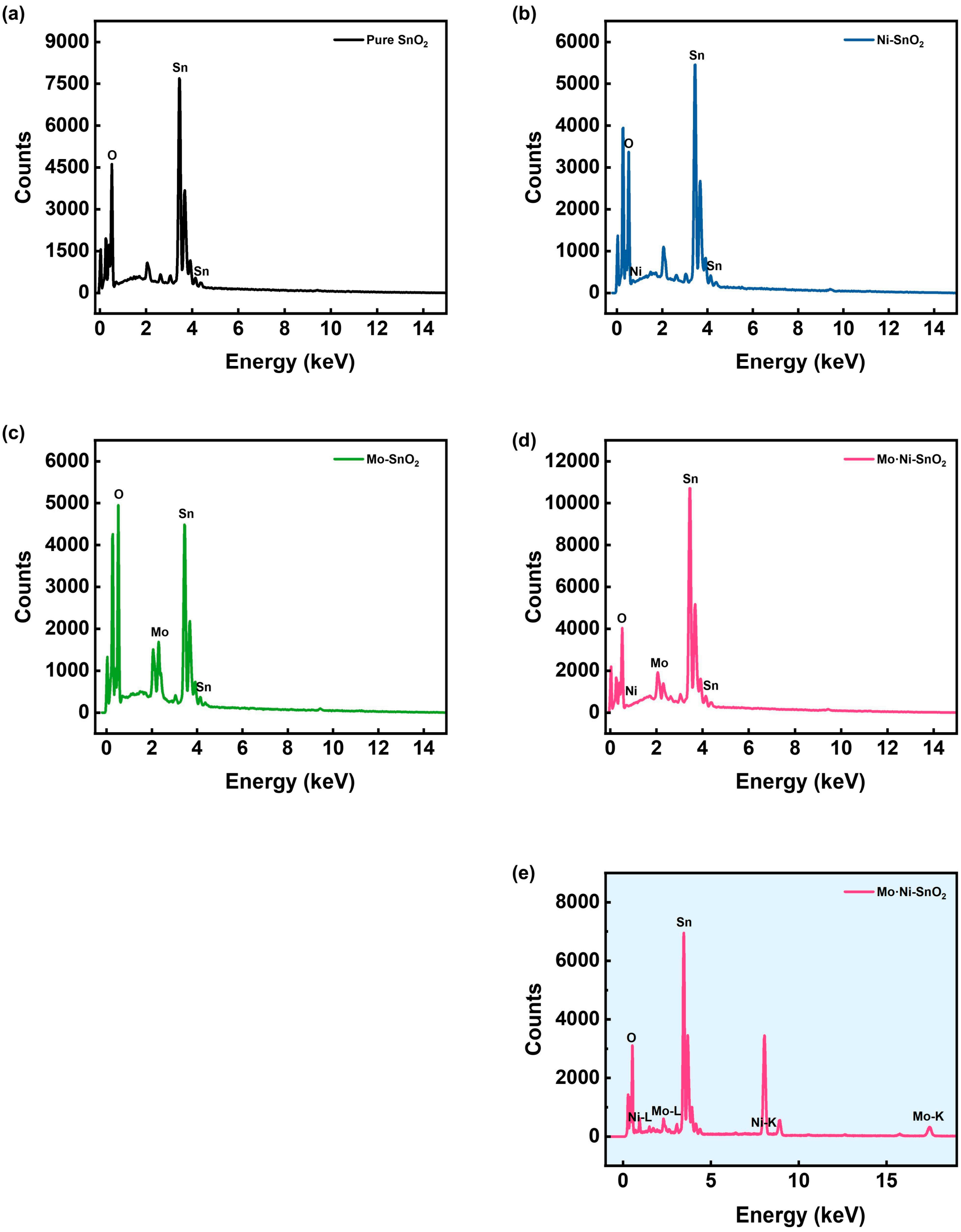

Figure S6 EDS spectrum with SEM of **(a)** Pure SnO_2, **(b)** Ni-SnO_2, **(c)** Mo-SnO_2, and **(d)** Mo·Ni-SnO_2; **(e)** EDS spectrum with HRTEM of Mo·Ni-SnO_2.

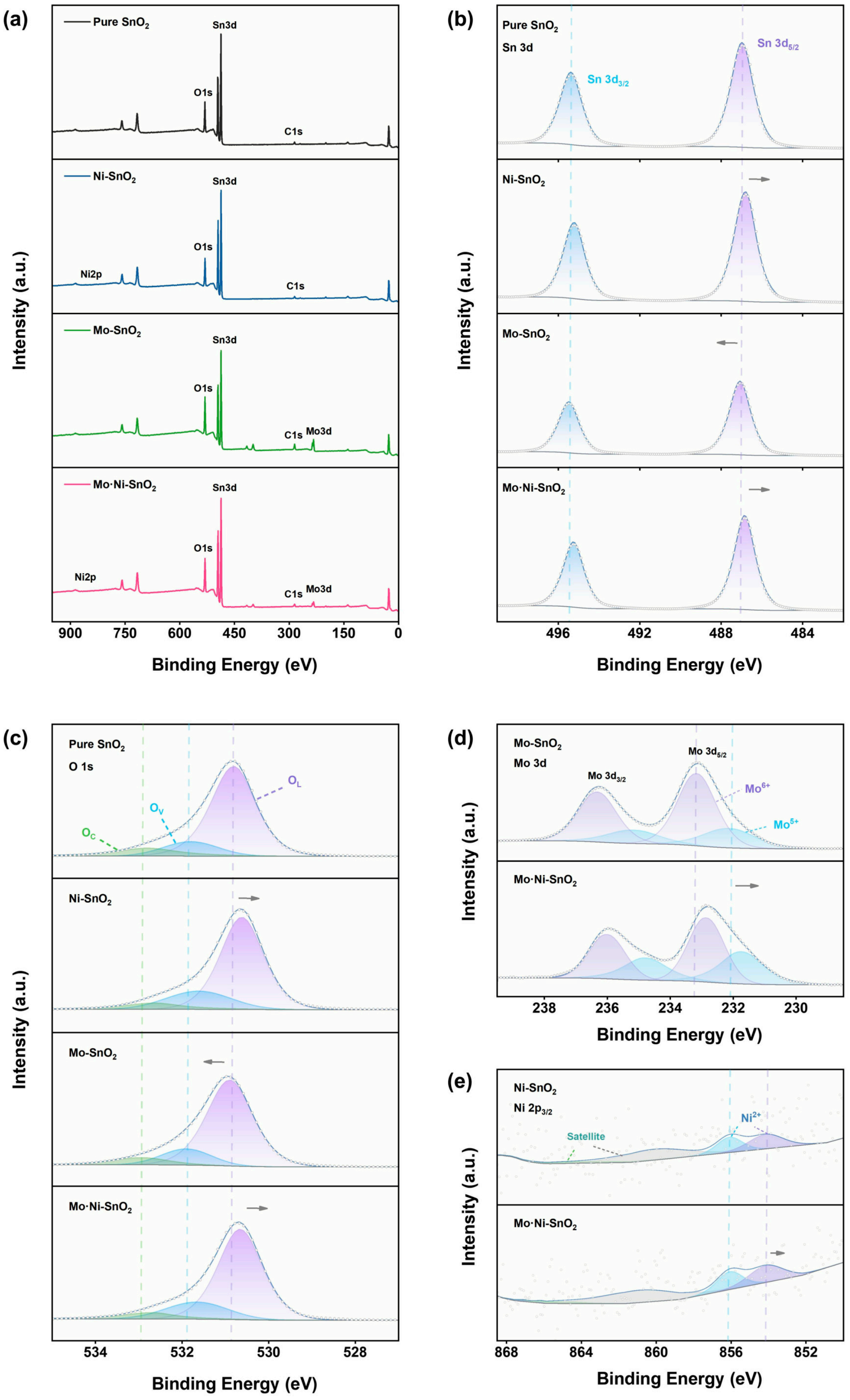

Figure S7 XPS spectra **(a)** Full scan of four materials (Pure SnO_2, Ni-SnO_2, Mo-SnO_2, and Mo·Ni-SnO_2), **(b)** Sn 3d of four materials, **(c)** O 1s of four materials, **(d)** Mo 3d of Mo-SnO_2, Mo·Ni-SnO_2, and **(e)** Ni $2p_{3/2}$ of Ni-SnO_2 and Mo·Ni-SnO_2.

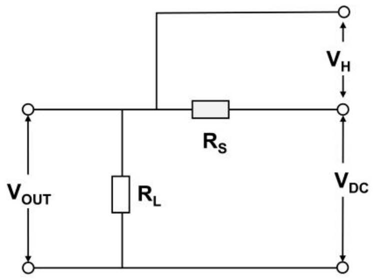

Figure S8 Simplified circuit diagram of the MEMS gas sensor test element.

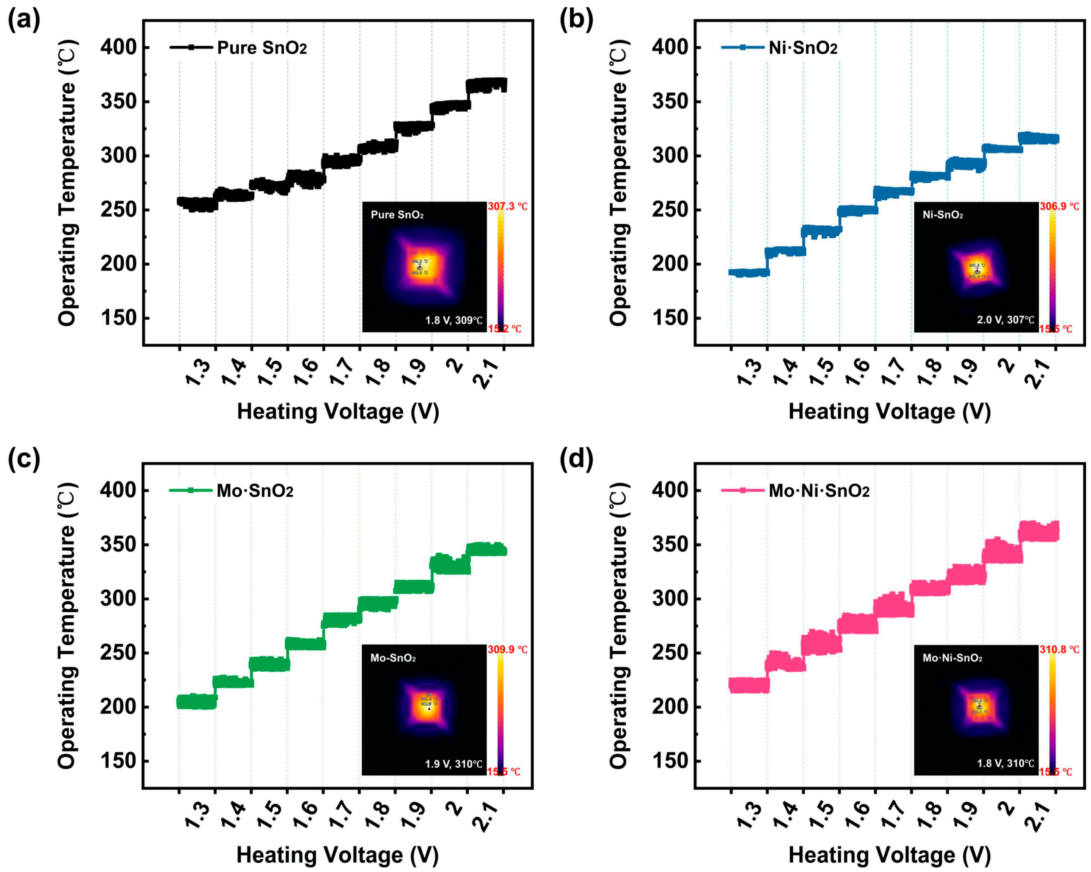

Figure S9 Relationship between the operating temperature and heating voltage of different MEMS sensors (Inset is the thermal imaging on the micro-hotplate at a certain heating voltage).

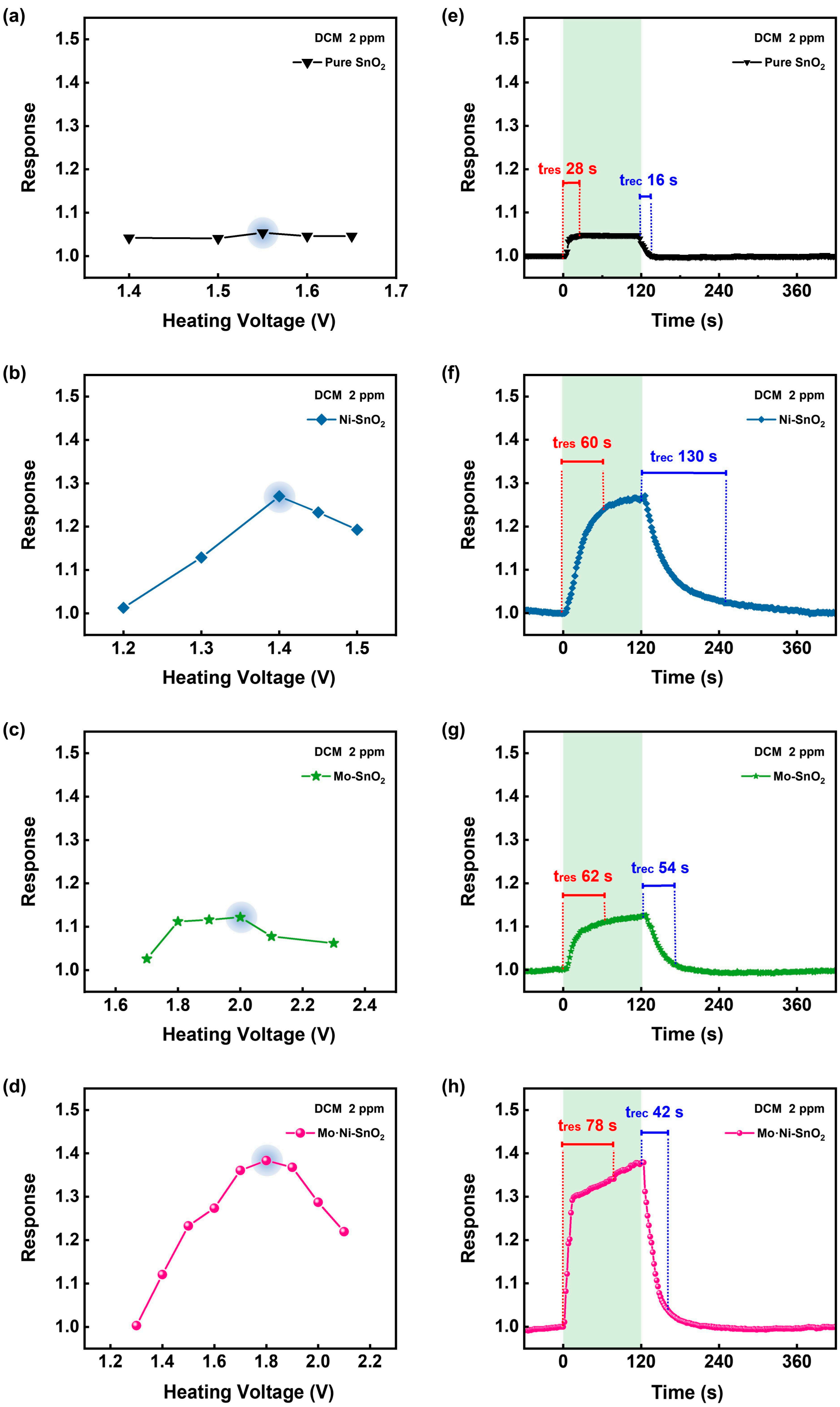

Figure S10 (a-d) Relationship between response and heating voltage and **(e-h)** Dynamic response curves of 4 MEMS sensors to 2 ppm DCM at the optimal heating voltage.

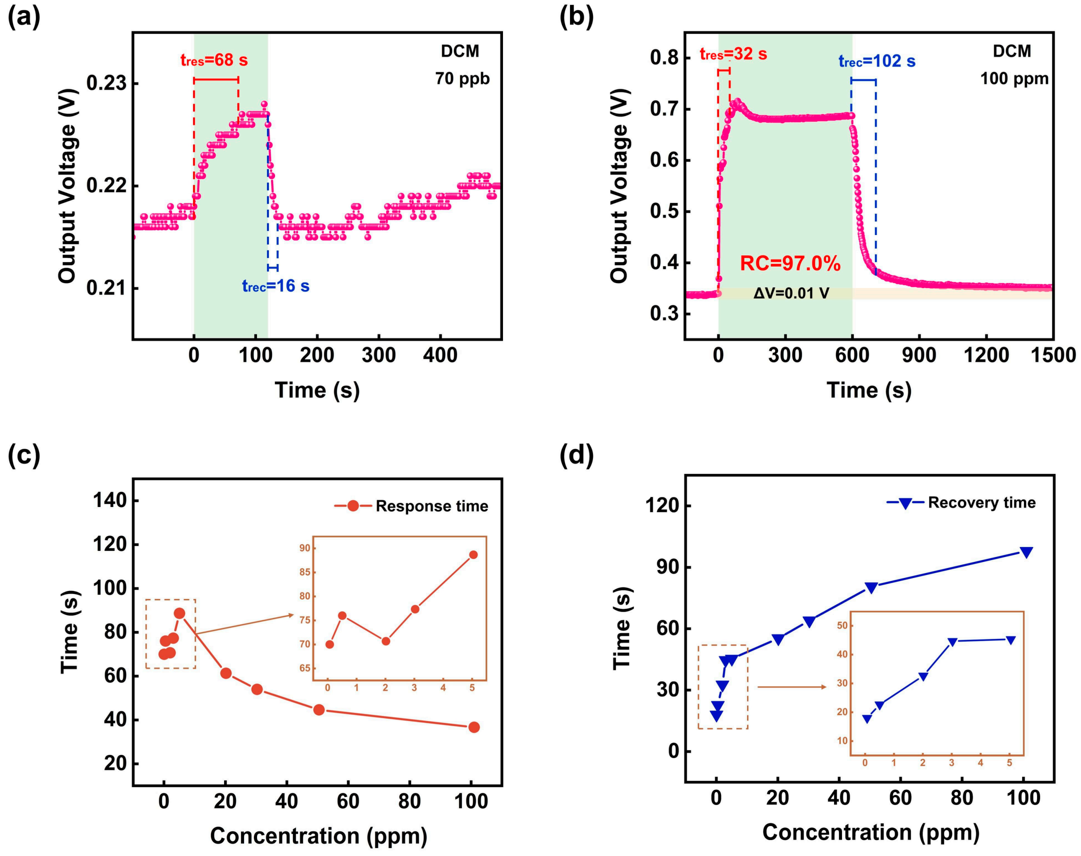

Figure S11 **(a)** Response and recovery time of MEMS sensor Mo·Ni-SnO_2 to 70 ppb DCM, **(b)** Response time, recovery time, and recovery capability of MEMS sensor Mo·Ni-SnO_2 to 100 ppm DCM, **(c)** Relationship between response time and concentration of DCM (70 ppb to 100 ppm), and **(d)** Relationship between recovery time and concentration of DCM (70 ppb to 100 ppm).

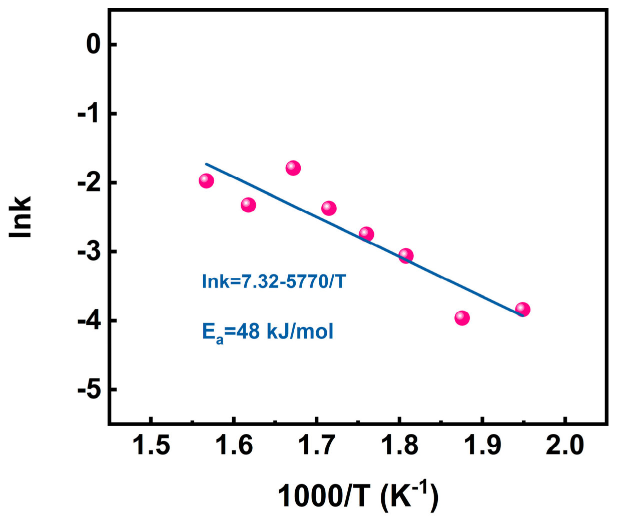

Figure S12 Temperature dependence of linear fitted plots of lnk in the presence of 2 ppm DCM using Mo·Ni-SnO_2 as sensing materials.

The inset of Figure 3d is the curve of output voltage (V_{OUT}) to time when the MEMS sensor Mo·Ni-SnO_2 exposed to DCM under 50 ppm, i.e., ln(C)=3.9. A non-linear fitting of this curve was carried out by $V_{OUT} = V_s - (V_s - V_a)e^{-kt}$, which can be transformed into $V_s - V_{OUT} = (V_s - V_a)e^{-kt}$, where V_a and V_s are the stable V_{OUT} when sensors are exposed to ambient air and DCM, respectively. k is the kinetic rate constant related to the change in the output voltage.

Similarly, the output voltage of the sensor when exposed to 2 ppm of DCM at different operating temperatures were fitted, and the corresponding kinetic rate constants k were obtained. Then a scatter plot of $\ln k$ versus 1000/T was obtained as Figure S12. Subsequently, $\ln k = 7.32 - 5770/T$ was obtained by performing a linear fit on the scatters. According to the Arrhenius equation $k = A e^{-E_a/RT}$, where the molar gas constant R is 8.314 $J \cdot mol^{-1} \cdot K^{-1}$, the value of the activation energy E_a was calculated as approximately 48 $kJ \cdot mol^{-1}$.

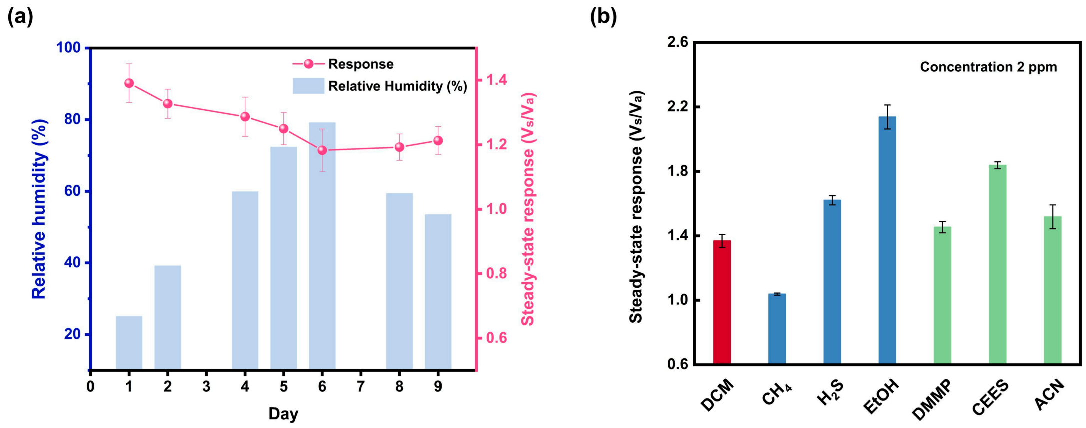

Figure S13 Stability and selectivity of MEMS sensor Mo·Ni-SnO_2 to DCM. **(a)** The stability test results of response of the sensor during 9 days at 310 ℃ in an environment where the relative humidity fluctuates greatly and **(b)** Selectivity investigation of the sensor towards 2 ppm DCM, CH_4, H_2S, EtOH, dimethyl methylphosphonate (DMMP), 2-chloroethyl ethyl sulfide (CEES), and acetonitrile (ACN) at 310℃; here DMMP, CEES, and ACN are the simulant agents of chemical warfare agents sarin, mustard gas, and hydrogen cyanide, respectively.

Section S3 Equations

S3.1 Equations of response (R), sensitivity (S), steady-state response (SR), and recovery capability (RC)

$$\mathrm{Response} = \frac{V_g}{V_a} \tag{S1}$$

$$S = \left|1 - \frac{V_g}{V_a}\right| \times 100\% \tag{S2}$$

$$SR = \frac{V_s}{V_a} \tag{S3}$$

$$RC = (1 - \frac{|\Delta V|}{V_a}) \times 100\%,\ \Delta V = V_a' - V_a \tag{S4}$$

S3.2 Calculation of the chemical potential μ_e for the electrons in conductor

According to the second law of thermodynamics, one can get

$$\mu = \left(\frac{\partial A}{\partial n}\right)_{T,V} \tag{S5}$$

$$A = U - TS \tag{S6}$$

$$\left(\frac{\partial A}{\partial n}\right)_{T,V} = \left(\frac{\partial U}{\partial n}\right)_{T,V} - T\left(\frac{\partial S}{\partial n}\right)_{T,V} \tag{S7}$$

$$\mu = \Delta U_m - T\Delta S_m \tag{S8}$$

$$\Delta U_m = \int_0^T C_V^e dT \tag{S9}$$

$$\Delta S_m = \int_0^T \frac{C_V^e}{T} dT \tag{S10}$$

Since the specific heat capacity of the electron is a function of the temperature, as[7]

$$C_V^e = \frac{\pi^2 R k_B}{2E_F} T \approx 10^{-4} RT \qquad \textbf{(S11)}$$

$$\mu_e(\mathrm{T}) = \Delta U_m - T\Delta S_m = \int_0^T C_V^e dT - T\int_0^T \frac{C_V^e}{T} dT = -5\times10^{-5} RT^2 \qquad \textbf{(S12)}$$

Therefore, the chemical potential of electrons at room temperature should be extremely less than one unit of electro voltage (eV), as the following calculation.

$$\mu_e(300K) = -5\times10^{-5}\times8.314\times300^2 = -37.4\ \mathrm{J/mol} = -3.74\times10^{-4}\ \mathrm{eV} \qquad \textbf{(S13)}$$

Reference

[1] W S Lee, S C Lee, S J Lee, et al. The sensing behavior of SnO_2 -based thick-film gas sensors at a low concentration of chemical agent simulants[J]. Sensors and Actuators B: Chemical, 2005, 108(1-2): 148-153.

[2] K H Yun, K Y Yun, G Y Cha, et al. Gas sensing characteristics of ZnO-doped SnO_2 sensors for simulants of the chemical agents[J]. Materials Science Forum, 2005, 486-487: 9-12.

[3] N J Choi, J H Kwak, Y T Lim, et al. Classification of chemical warfare agents using thick film gas sensor array[J]. Sensors and Actuators B: Chemical, 2005, 108(1-2): 298-304.

[4] S C Lee, H Y Choi, S J Lee, et al. Novel SnO_2-based gas sensors promoted with metal oxides for the detection of dichloromethane[J]. Sensors and Actuators B: Chemical, 2009, 138(2): 446-452.

[5] S C Lee, S Y Kim, W S Lee, et al. Effects of textural properties on the response of a SnO_2-based gas sensor for the detection of chemical warfare agents[J]. Sensors, 2011, 11(7): 6893-6904.

[6] H M Aliha, A A Khodadadi, Y Mortazavi. The sensing behaviour of metal oxides (ZnO, CuO and Sm_2O_3) doped-SnO_2 for detection of low concentrations of chlorinated volatile organic compounds[J]. Sensors and Actuators B: Chemical, 2013, 181: 637-643.

[7] J J Quinn, K su Yi. Solid State Physics: Principles and Modern Applications[M]. 2nd ed. 2018. Cham: Springer, 2018.
